# Protein degradation: expanding the toolbox to restrain cancer drug resistance

**DOI:** 10.1186/s13045-023-01398-5

**Published:** 2023-01-24

**Authors:** Hui Ming, Bowen Li, Jingwen Jiang, Siyuan Qin, Edouard C. Nice, Weifeng He, Tingyuan Lang, Canhua Huang

**Affiliations:** 1 West China School of Basic Medical Sciences and Forensic Medicine, and State Key Laboratory of Biotherapy and Cancer Center, West China Hospital, Sichuan University, and Collaborative Innovation Center for Biotherapy, Chengdu, 610041 People’s Republic of China; 2 Department of Biochemistry and Molecular Biology, Monash University, Clayton, VIC 3800 Australia; 3 Institute of Burn Research, Southwest Hospital, State Key Laboratory of Trauma, Burn and Combined Injury, Chongqing Key Laboratory for Disease Proteomics, Army Military Medical University, Chongqing, 400038 China; 4 Department of Gynecologic Oncology, Chongqing University Cancer Hospital & Chongqing Cancer Institute & Chongqing Cancer Hospital, Chongqing, 400030 People’s Republic of China; 5 Reproductive Medicine Center, The First Affiliated Hospital of Chongqing Medical University, Chongqing, 400042 People’s Republic of China

**Keywords:** E3 ligase, DUBs, Chaperone-mediated autophagy, Protein degradation, Drug resistance

## Abstract

Despite significant progress in clinical management, drug resistance remains a major obstacle. Recent research based on protein degradation to restrain drug resistance has attracted wide attention, and several therapeutic strategies such as inhibition of proteasome with bortezomib and proteolysis-targeting chimeric have been developed. Compared with intervention at the transcriptional level, targeting the degradation process seems to be a more rapid and direct strategy. Proteasomal proteolysis and lysosomal proteolysis are the most critical quality control systems responsible for the degradation of proteins or organelles. Although proteasomal and lysosomal inhibitors (e.g., bortezomib and chloroquine) have achieved certain improvements in some clinical application scenarios, their routine application in practice is still a long way off, which is due to the lack of precise targeting capabilities and inevitable side effects. In-depth studies on the regulatory mechanism of critical protein degradation regulators, including E3 ubiquitin ligases, deubiquitylating enzymes (DUBs), and chaperones, are expected to provide precise clues for developing targeting strategies and reducing side effects. Here, we discuss the underlying mechanisms of protein degradation in regulating drug efflux, drug metabolism, DNA repair, drug target alteration, downstream bypass signaling, sustaining of stemness, and tumor microenvironment remodeling to delineate the functional roles of protein degradation in drug resistance. We also highlight specific E3 ligases, DUBs, and chaperones, discussing possible strategies modulating protein degradation to target cancer drug resistance. A systematic summary of the molecular basis by which protein degradation regulates tumor drug resistance will help facilitate the development of appropriate clinical strategies.

## Background

For several decades, the rapid development of novel therapeutic strategies has significantly contributed to the decline in the mortality in patients with cancer [1]. However, the clinical therapeutic outcome of late-stage cancer is still far from satisfactory, where drug resistance is considered an essential molecular mechanism [2]. These molecular mechanisms of therapeutic tolerance have been studied intensively and can be categorized into pharmacokinetic and pharmacodynamic resistance pathways [3, 4]. The pharmacokinetic resistance mechanisms include increased drug efflux, reduced drug uptake, alterations in drug metabolism, and drug sequestration, which lead to decreased intracellular drug concentrations [5, 6]. Activation of DNA repair, alterations in drug targets, evasion of cellular death pathways, and remodeling of the tumor microenvironment account for pharmacodynamic resistance. Furthermore, classified according to cause, drug resistance can be divided into two categories: innate and acquired resistance [7]. Innate resistance causes chemotherapy to be ineffective before drug treatment as a result of resistance-mediating factors that preexist in the bulk of tumor cells [8]. On the other hand, acquired chemoresistance can be developed under the stress of treatment, which endows cancer cells with acquired epigenetic alterations for enhanced survival ability [9–11]. In addition to the regulation of gene transcriptional and posttranscriptional modification [12, 13], protein degradation has received increasing attention due to its direct and rapid regulation.

**Fig. 1:**
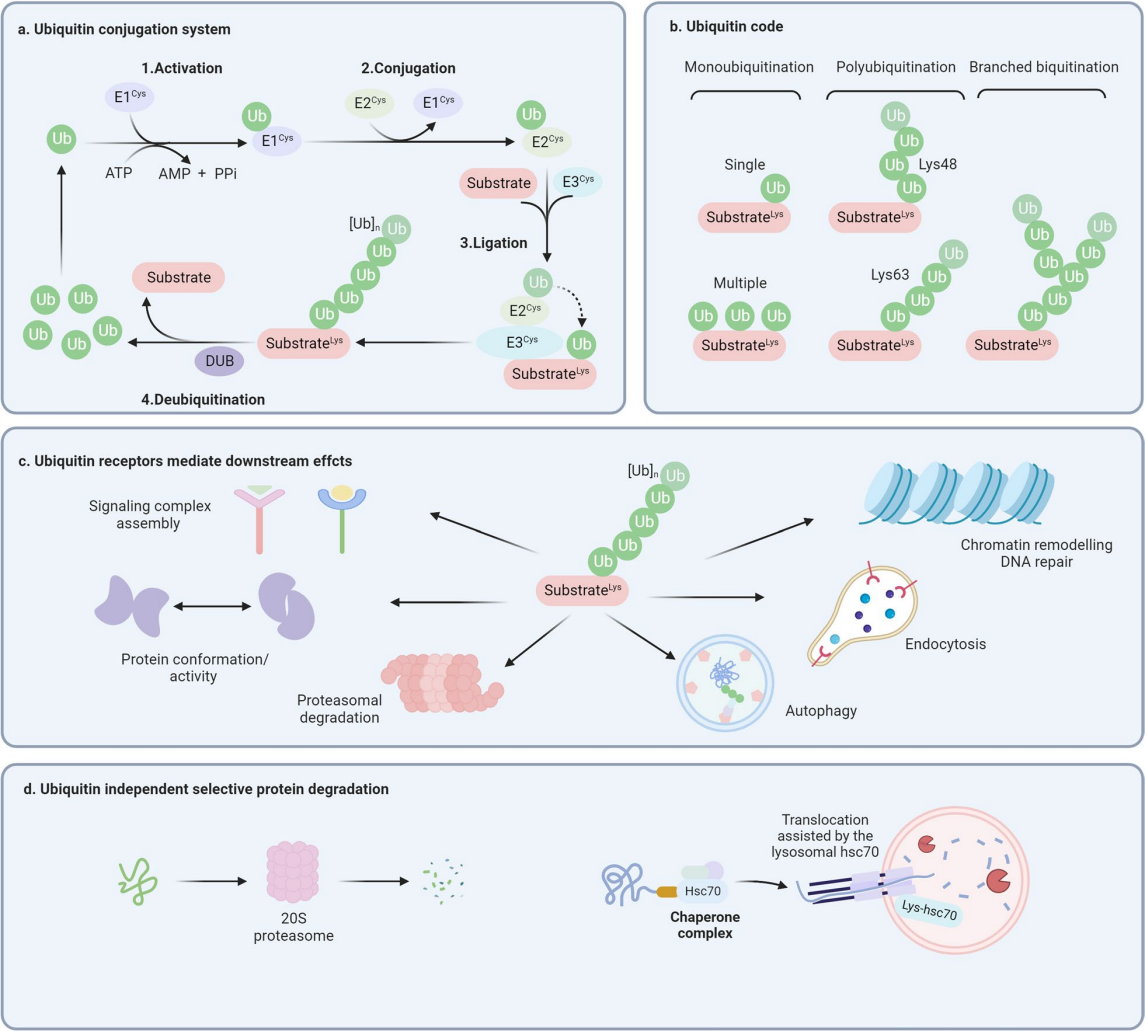
Ubiquitin-dependent and independent molecular mechanisms of protein degradation. **a** Ubiquitin conjugation system consists of ubiquitin-activating enzyme (E1), ubiquitin-conjugating enzyme (E2), and ubiquitin ligase (E3). Deubiquitylating enzymes (DUB) can remove ubiquitin chains from substrates. **b** Ubiquitin modification can be categorized as monoubiquitination, polyubiquitination, and branched ubiquitination, where Lys48 and Lys63 polyubiquitination chains are closely related to protein degradation. **c** The biological functions of substrates, including signaling transduction, protein conformation change, chromatin remodeling, proteasomal degradation, and lysosomal degradation, are further determined by the ubiquitin receptors. **d** Ubiquitin-independent targeted protein degradation includes 20S proteasome-mediated protein degradation and chaperone-mediated autophagy

Protein homeostasis is essential for various cellular and organismal functions [14]. Proteins to be degraded usually contain specific recognition motifs termed “degrons,” including short amino acid sequences with (or without) specific posttranslational modifications (e.g., phosphorylation or glycosylation) [15], exposed amino acids (e.g., N-degrons and C-degrons) [16], and structural motifs [17]. After being identified, these proteins are usually conjugated with specific tags (e.g., ubiquitin) and degraded by proteolytic systems. Proteasomal proteolysis and lysosomal proteolysis are two major quality control systems responsible for the degradation of cellular proteins [18]. Generally, proteasomal proteolysis is a multi-enzyme process in which the covalent conjugation of ubiquitin to substrates induces degradation by the proteasome [19]. The most common mammalian proteasome involved in degradation is the 26S proteasome, consisting of a 20S core particle with proteolytic activity and two 19S cap subunits that recognize polyubiquitin tags on substrates [20]. With some exceptions, a small portion of proteins can interact with the 20S and be degraded through a ubiquitin-independent pathway [21]. Lysosomal proteolysis is a multistep lysosome-dependent degradation process whereby cytoplasmic constituents (e.g., protein aggregates, damaged organelles) are degraded through posttranslational modification (e.g., ubiquitin-dependent or chaperone-mediated mechanisms) [22]. The lysosomal degradation pathway can be mainly divided into autophagy and endocytosis according to the origin of the substrate from autophagosome or endosome, respectively [23]. Considered the most common lysosomal degradation pathway, autophagy can be categorized into macroautophagy (the most studied type of autophagy), microautophagy, and chaperone-mediated autophagy [24]. Substrates (e.g., specific proteins, protein aggregates, and organelles) with specific cargo signals (e.g., ubiquitin) can be recognized by autophagy receptors (e.g., p62, BNIP3), resulting in selective degradation [25]. Apart from ubiquitin-mediated selective autophagy, chaperone-mediated autophagy facilitates protein degradation by recognizing specific motifs (KFERQ-like motifs) [26]. The protein degradation process is widely adopted as a dynamic and self-regulating process for cellular quality control and a decision process for cells that discriminate between normal and malfunctioning proteins or subcellular structures [27] (Fig. 1).

With respect to the common recognition signal for both proteasomal and lysosomal degradation, ubiquitin (an 8.6 kD small regulatory protein) has been recognized as one of the essential posttranslational modifications governing protein degradation through both proteasomes and lysosomes [28]. Ubiquitination mainly involves three steps: ubiquitin-activating enzymes (E1s)-mediated activation, conjugating through ubiquitin-conjugating enzymes (E2s), and ligation executed by ubiquitin ligases (E3s). There exist at least 8 E1s [29], ~ 40 E2s [30], and ~ 600 E3s [31], among which E3s have the ability of substrate-specific recognition for precise regulation [32]. E3s can be categorized into three families, including homologous to the E6AP C-terminus (HECT), really interesting new gene (RING)-finger/UFD2 homology (U-box), and RING-between-RING (RBR) families [33]. In addition, ubiquitination can also be removed by deubiquitylating enzymes (DUBs), indicating that the ubiquitination system is finely regulated [34]. To date, the ~ 100 putative DUBs can be classified into two classes: cysteine proteases and metalloproteases [35]. The ubiquitination of a specific protein can be either a single ubiquitin or ubiquitin chains, where secondary ubiquitin proteins are linked to one of the lysine residues (K6, K11, K27, K29, K33, K48, K63) or N-terminal methionine (M1) [36]. The ubiquitin modification has a wide range of physiological functions, including ubiquitin-mediated degradation, ubiquitination signal transduction, chromatin remodeling, DNA repair, and protein identification [37]. Generally, proteins with K11 and K48 polyubiquitin modification tend to be degraded through the proteasome [38], and proteins with monoubiquitylation and K63 polyubiquitin modification are degraded through selective autophagy [39], which is probably due to the competitive binding of two ubiquitin-binding receptors, valosin-containing protein (VCP)/p97 and p62, to polyubiquitin chains in different degradation systems [40]. Recent studies suggest that there is a complementary and competitive relationship between the two degradation systems, and the regulatory mechanism remains to be further explored [41, 42]. In addition, the chaperone Hsc70 (also known as HSPA8) can directly bind substrates containing a KFERQ-like motif, thus mediating lysosomal degradation by interacting with lysosome-associated membrane protein type 2A (LAMP2A) [43]. Recent evidence suggests that critical regulators in selective protein degradation, including E3s, DUBs, and chaperones, are deregulated in multiple types of tumors, suggesting that studying the underlying molecular mechanisms may facilitate the identification of drug targets and the development of therapeutic regimens [44].

Compared with regulation at the transcriptional level, the protein degradation process seems to be a more rapid and direct mechanism for cancer drug resistance [45]. However, studies on endogenous degradation processes and their interplay with drug resistance are still limited due to the complex interactions between endogenous and exogenous signals [46–48]. Identifying molecular mechanisms is of significance for finding potential clinically meaningful targets and developing novel therapeutic approaches. In this review, we aim to systematically describe the molecular mechanisms between protein degradation and drug resistance, the regulatory mechanism between E3s/DUBs/chaperones and drug resistance-related proteins and their potential clinical application value. In addition, we will outline the emerging approaches targeting protein degradation in recent years and discuss their current use and application prospects.

## Protein degradation-mediated aberrant drug transport and metabolism

Cancer cell pharmacokinetic resistance leads to reduced intracellular drug concentrations, including increased drug efflux, reduced drug uptake, and altered drug metabolism. Recently, multiple lines of evidence have demonstrated that protein degradation participates in the modulation of therapeutic resistance by regulating drug transport and metabolism [49].

### Drug efflux modulated by protein degradation

**Fig. 2:**
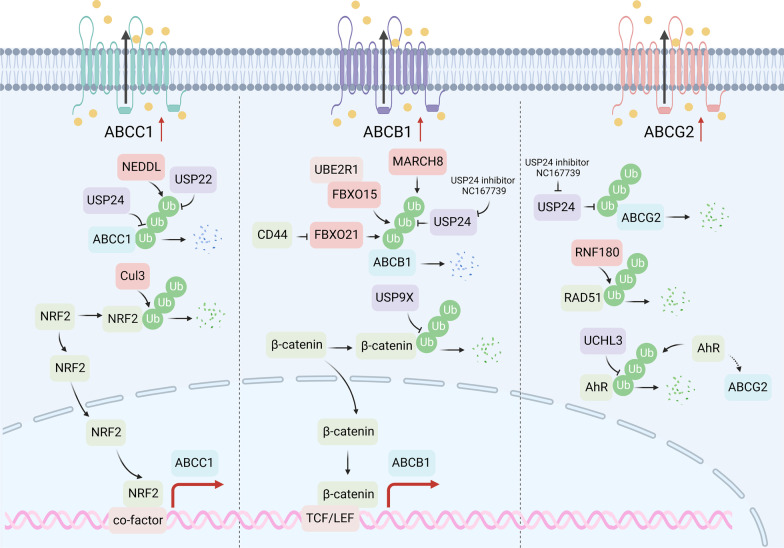
Protein degradation regulates drug efflux. ABCC1, ABCB1, and ABCG2 are the three main drug transporters. NEDDL ligases the ubiquitin chain to ABCC1, while USP22 and USP24 eliminate the ubiquitin chain. CUL3 regulates ABCC1 expression by modulating the degradation of NRF2. MARCH8, FBXO15, and FBXO21 ligase the ubiquitin chain on ABCB1, and USP24 acts as the DUB. USP24 is the DUB of ABCG2. RNF180 and UCHL3 indirectly regulate the expression of ABCG2

Adequate intertumoral drug exposure is a prerequisite for effective drug treatment. Once chemotherapeutic agents have reached cancer cells, passage across the plasma membrane represents the first possible impediment to effective drug delivery into cancer cells. However, when tumor cells highly express drug efflux receptors, they can sustain a low intracellular drug concentration during drug treatment, thereby promoting tumor resistance [50]. Most notably, among the 48 ATP-binding cassette (ABC) transporter family proteins, three members have been implicated as multi-drug efflux pumps capable of conferring multi-drug resistance (MDR), including multi-drug resistance protein 1 (MDR1, also known as ABCB1 and P-glycoprotein), MDR-associated protein 1 (MRP1, or ABCC1) and breast cancer resistance protein (BCRP, or ABCG2) [51]. These proteins serve as cell membrane pumps that effectively block or limit the access of drugs into cancer cells, resulting in poor therapeutic outcomes across many different cancers [52]. Interestingly, recent studies indicate that protein degradation participates in the regulation of ABC family protein expression, directly on selective degradation or indirectly via upstream signaling pathways, thus influencing the drug efflux of tumor cells (Fig. 2).

#### ABCB1

ABCB1 is the most in-depth studied ABC transporter, participating in drug resistance in multiple types of tumors [53]. Several E3s and DUBs are involved in the regulation of ABCB1 degradation through direct or indirect mechanisms. For example, evidence shows that FBXO15 can interact with ABCB1 and facilitate its ubiquitination [54]. The downregulation of ABCB1 expression by ubiquitin-conjugating enzyme E2 R1 (UBE2R1) and FBXO15-mediated ubiquitination boosted sensitivity to vincristine and doxorubicin [55]. Additionally, the F-box protein family member FBXO21 is another subunit of SCF ubiquitin E3 ligases, which function in phosphorylation-dependent ubiquitination degradation [56]. FBXO21 is involved in the proteasome-mediated degradation of ABCB1, resulting in the attenuation of MDR. However, the hyaluronic acid (HA) receptor CD44 was found to impair FBXO21-directed degradation of ABCB1, leading to the enhancement of MDR in a CD44 phosphorylation-dependent manner [57]. Therefore, targeting CD44 could represent a potential therapeutic strategy to overcome drug resistance for cancer cells overexpressing both CD44 and ABCB1. MARCH8, a member of the membrane-associated RING-CH (MARCH) protein family, is a transmembrane E3 ligase that targets glycoproteins for lysosomal destruction [58], whose downregulation modulates the drug sensitivity of breast cancer cells [59]. Mechanistically, MARCH8 can interact with ABCB1 and promote its ubiquitination and degradation, thereby antagonizing ABCB1-mediated drug efflux [60].

In addition, DUBs are also found to participate in drug resistance by regulating the ABC transporter directly or indirectly. For example, USP24 positively regulates drug resistance partially by stabilizing ABC transporters, including ABCB1, ABCG1, and ABCC1, to pump out drugs from cancer cells [61]. USP9X upregulates the expression of ABCB1 and MRP2 by stabilizing *β*-catenin, thus affecting cisplatin resistance in nasopharyngeal carcinoma (NPC) cells [62].

#### ABCC1

The first identified ABCC family protein, ABCC1, is regulated by E3s and DUBs. Neural precursor cell expressed developmentally downregulated gene 4-like (NEDD4L) is predicted as an E3 ligase to regulate the ubiquitination of ABCC1 in lung squamous cell carcinoma (LSCC) [63]. Additionally, ABCC1 is found to be indirectly upregulated by the silenced CUL3 KEAP1 E3 ligase in breast cancer, stabilizing nuclear factor erythroid 2-related factor 2 (NRF2), and subsequently increases NRF2-regulated *ABCC1* transcription, which exhibits resistance to both doxorubicin and paclitaxel [64]. Likewise, inactivated ubiquitin E3 ligase Trc8 accounts for a lower ubiquitination rate of 3-hydroxy-3-methylglutaryl-coenzyme A reductase (HMGCoAR) and higher cholesterol synthesis in the plasma membrane, which favors the activity of ABC transporters and limits the intracellular accumulation of chemotherapeutic drugs [65].

Notably, upregulation of USP22 increases ABCC1 expression and subsequently contributes to sorafenib resistance in hepatocellular carcinoma (HCC) cells, suggesting that USP22 may serve as a therapeutic target for surmounting sorafenib resistance [66]. Another study revealed that sorafenib and USP22 shRNA co-delivery system exhibits remarkedly strong antitumor efficiency by suppressing ABCC1 in the process of synergetic HCC therapy [67]. Moreover, USP22 directly interacts with sirtuin 1 (SIRT1) and positively regulates SIRT1 protein expression, leading to activation of the SIRT1/AKT/ABCC1 pathway and MDR of hepatocellular carcinoma [68]. USP22 is also found to indirectly promote the expression of MDR-related genes through upregulation of AKT and subsequently activation of the PI3K pathway in HCC [69].

#### ABCG2

In the case of ABCG2, a recent study revealed that RNF180 increases the sensitivity of triple-negative breast cancer cells to gefitinib by degrading RAD51 and downregulation of efflux transporters, including ABCG2, ABCC1, and ABCB1 [70]. USP24 increases the levels of ABC transporters ABCB1, ABCG2, and ezrin to enhance the pumping of taxol from cancer cells. Thus, employing NC167739, a USP24 inhibitor, can effectively block drug resistance during chemotherapy [61]. Ubiquitin C-terminal hydrolase L3 (UCHL3) stabilizes aryl hydrocarbon receptor (AhR) by its deubiquitylation and subsequently increases ABCG2 to promote stem-like properties in lung cancer [71].

### Drug metabolism and protein degradation

**Fig. 3:**
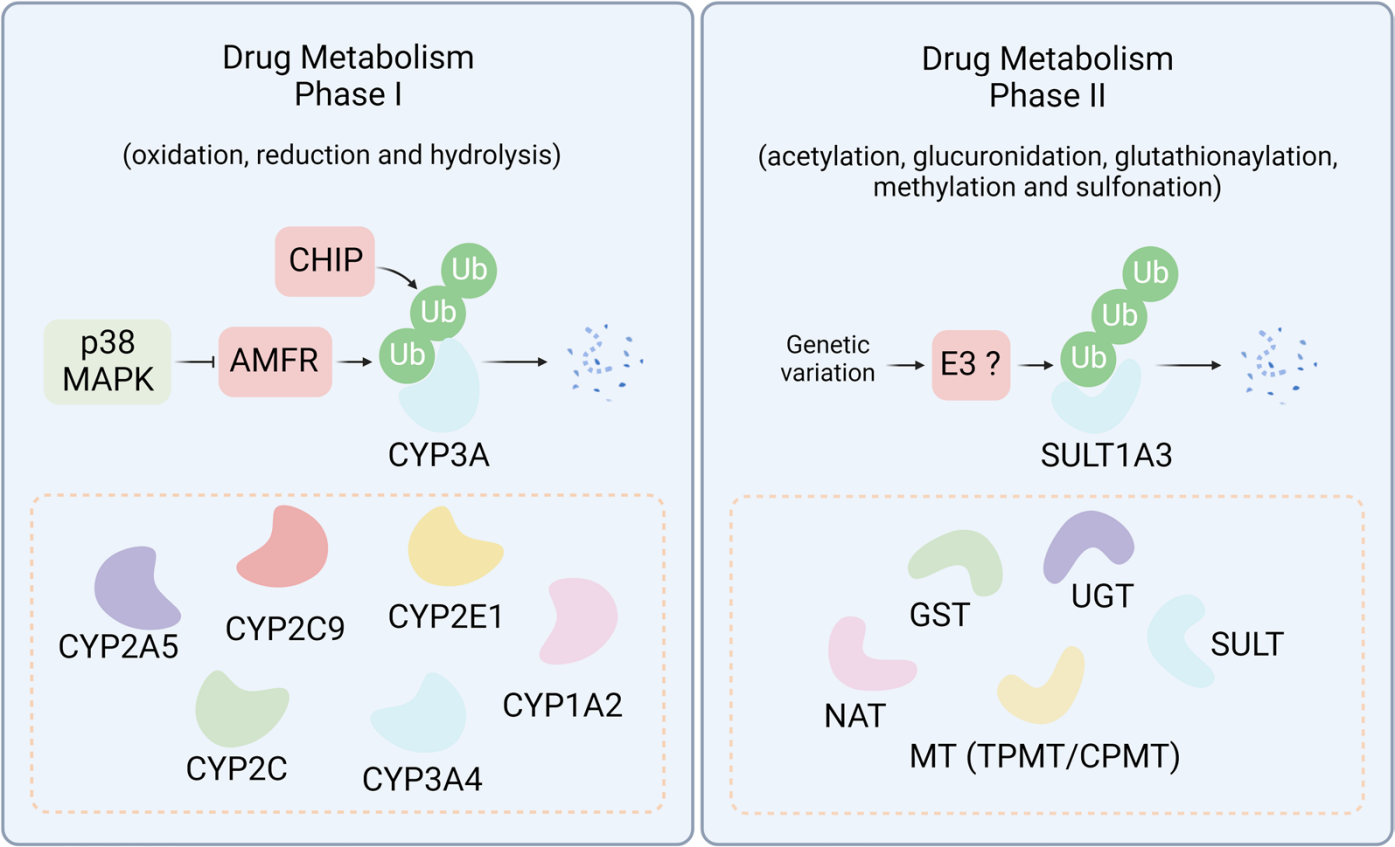
Protein degradation modulates drug metabolism. Drug metabolism can be categorized into Phase I (oxidation, reduction, and hydrolysis) and Phase II (posttranslational modification), requiring different classes of drug metabolic enzymes. In Phase I drug metabolism, CHIP and AMFR can ubiquitinate the CYP3A family for degradation. In Phase II, SULT1A3 is regulated by ubiquitin-dependent protein degradation

Drug inactivation is another upstream resistance mechanism [72] that involves two distinctive pathways, including Phase I metabolism (oxidation, reduction, and hydrolysis reactions) and Phase II metabolism (binding reactions) [73]. Notably, the key metabolic enzymes responsible for Phase I metabolism are cytochrome P450 enzymes (CYP450), such as the CYP3A4, CYP1A1, CYP1A2, and CYP2 families [74]. The main enzymes involved in Phase II metabolism include UDP-sulfotransferases (SULTs), glucuronosyltransferases (UGTs), glutathione S-transferases (GSTs), N-acetyltransferases (NATs), and various methyltransferases (MTs) [75]. Recent studies indicate that targeted protein degradation participates in the modulation of drug metabolism, thereby affecting cancer drug resistance (Fig. 3).

E3 ligase autocrine motility factor receptor (AMFR, or GP78) plays a pivotal role in metastasis, tumor progression, and recurrence [76]. Liver-specific genetic ablation of AMFR results in concurrent stabilization of several therapeutically relevant P450s by inhibiting endoplasmic reticulum-associated degradation (ERAD), including CYP2A5, CYP2C, CYP3A, and CYP2E1, which leads to corresponding enhancement of their drug-metabolizing capacities [77]. CYP3A4 is the most abundant P450 in the human liver and is responsible for the metabolism of over 50% of clinically relevant drugs [78]. Both UBC7/gp78 and UbcH5a/C terminuses of HSC70-interacting protein (CHIP) are involved in CYP3A4 ERAD, suggesting that ERAD-associated E3 ligases may influence drug metabolism by regulating the physiological CYP3A expression and its function [79, 80]. Intriguingly, endoplasmic reticulum-related protein quality control can be either proteasome or lysosome-dependent, where the regulatory mechanisms still need further elucidation [81]. Another study indicates that acetaminophen and salicylate derivatives could decrease the expression of AMFR protein through p38 MAPK activation, resulting in the inhibition of CYP3A protein degradation and a subsequent increase in enzymatic activity [82]. In addition, the detoxification-related enzyme sulfotransferase 1A3 (SULT1A3) is found to be degraded much more rapidly by a ubiquitin–proteasome system-dependent process in case of genetic variation [83].

## Degradation of critical proteins in DNA damage repair-mediated drug resistance

**Fig. 4:**
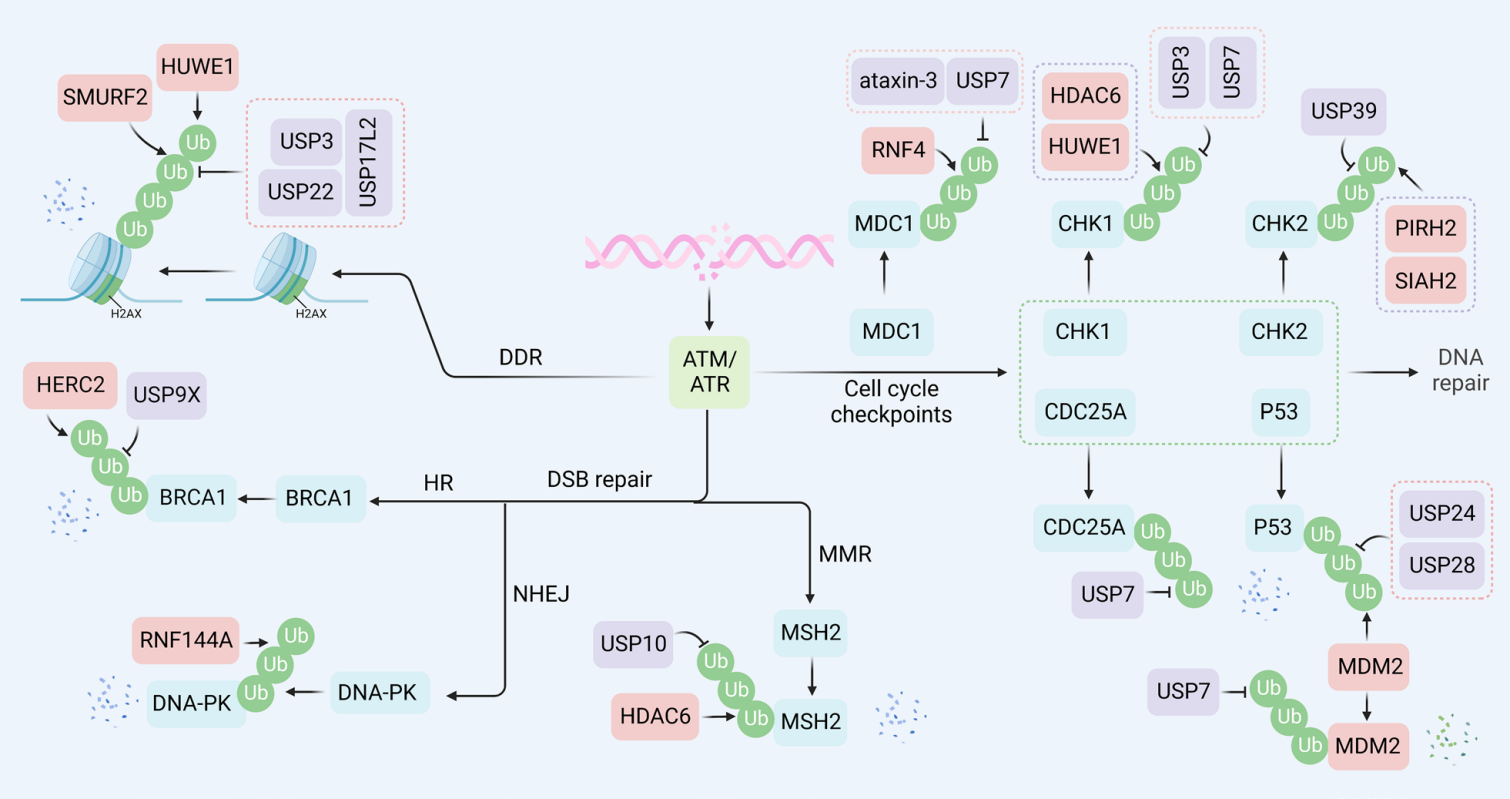
Regulatory functions of ubiquitination in DNA repair. E3 ligases usually participate in DNA damage response and DNA double-strand repair through degradation-dependent function. Specifically, during DNA damage response, H2AX can be degraded through SMURF2 and HUWEI-mediated ubiquitination, and USP3, USP22, and USP17L2 can eliminate the ubiquitin chains. During homologous recombination, BRCA1 can be degraded by HERC2 and stabilized by USP9X. In the NHEJ process, XRCC4 is ubiquitinated by FBXW7 for degradation. DNA-PK is degraded through RNF144A-mediated ubiquitination. During mismatch repair, HDAC6 ubiquitinates MSH2 for further protein degradation, which can be reversed by USP10. In the regulation of cell cycle check points, RNF4 promotes ubiquitination of MDC1, and ataxin-3 and USP7 deubiquitinate MDC1, therefore controlling the stability of MDC1. MDM2 facilitates the ubiquitinoylation of p53 for degradation, while USP4, USP24, USP7, and USP28 remove the ubiquitin chain. USP7 stabilizes CDC25A. PIRH2 and SIAH2 promote ubiquitin-dependent degradation of CHK2, and USP39 eliminates the ubiquitin chains. HDAC6 and HUWEI induce CHK1 ubiquitination, while USP3 and USP7 deubiquitinate the CHK1

The anticancer activity of most chemotherapy drugs relies on the induction of DNA damage in tumor cells with defective DNA repair, either directly or indirectly [84, 85]. For example, platinum-based drugs are especially used for testicular cancer, which directly form DNA adducts and lead to intrastrand and interstrand cross-links [86]. Anthracyclines induce DNA damage by inhibiting DNA topoisomerases and producing oxygen radicals indirectly [87]. Therefore, cancer cells may achieve drug resistance to DNA-damaging reagents through cell cycle arrest or DNA damage repair [88, 89]. The DNA damage response (DDR) system involves several stages, including damage sensing, signaling transduction, and damage repair [90]. Growing evidence suggests that DUB and E3 dysregulation promotes cancer cell resistance to DNA-damaging agents by mediating the ubiquitination of many proteins involved in DNA repair-associated pathways (Fig. 4).

### DNA damage response

H2AX is a variant of histone H2A. Phosphorylation of H2AX at Ser139 (*γ*H2AX) and subsequent ubiquitination of H2AX play a critical role in DNA damage repair, which promotes chromosome remodeling and recruitment of DNA repair enzymes [91].

The ubiquitin ligase RNF8 initiates the ubiquitination of H2AX, and RNF168 amplifies the ubiquitination response, which triggers the recruitment of p53-binding protein 1 (53BP1) and Rap80/BRCA1 to the lesion [92, 93]. RNF168 also interacts with another E3 ubiquitin ligase, SMURF2, to modulate the stability of H2AX in glioblastoma cells. Mechanistically, suppression of protein arginine methyltransferase 5 (PRMT5) attenuates the expression of RNF168 in methylthioadenosine phosphorylase (MTAP)-deficient glioblastoma cells, contributing to the destabilization of H2AX by SMURF2. These findings reveal that RNF168 and SMURF2 serve as stabilizers and destabilizers of H2AX, respectively, via the PRMT5-RNF168-SMURF2 signaling cascade [94]. In addition, RNF168 was found to mediate H2AX polyubiquitination and degradation under chronic oxidative stress, which provides new insights into the ROS-mediated regulation of H2AX turnover and is indicative of the therapeutic efficiency and survival of triple-negative breast cancer (TNBC) patients [95]. Moreover, H2AX can also be degraded via polyubiquitination mediated by HUWE1. ATM kinase, the sirtuin protein 6 (SIRT6), and the chromatin remodeler SNF2H mediate transient H2AX stabilization by blocking HUWE1 to allow *γ*H2AX foci formation when cells are damaged [96].

Given the importance of H2AX ubiquitination in triggering the DDR, several hydrolases have been shown to regulate the ubiquitination cascade directly or indirectly. Ubiquitin-specific peptidase 22 (USP22) deubiquitinates and phosphorylates H2AX to enhance DNA damage repair and induce cisplatin resistance in lung adenocarcinoma [97]. Ubiquitin hydrolase Dub3, also known as USP17L2, counteracts the H2AX E3 ligases RNF8 and RNF168, resulting in decreases in the DNA damage-induced monoubiquitination of H2AX. Importantly, Dub3 overexpression abrogates focus formation of 53BP1 and breast cancer type 1 susceptibility protein (BRCA1) in response to genotoxic stress [98]. Similarly, USP3 removes Ub at lysine 13 and 15 of H2A and *γ*H2AX, as well as 118 and 119 of H2AX in response to DNA damage, counteracting RNF168- and RNF8-mediated ubiquitination and impairing the accumulation of the downstream repair factors BRCA1 and 53BP1 at the damage sites [99].

Altogether, these results indicate that regulating E3s or DUBs might be a mechanism to correct DDR and to allow checkpoint recovery.

### Cell cycle checkpoints

Mediator of DNA damage checkpoint protein 1 (MDC1) is a vital modular phosphoprotein in controlling proper DDR and maintaining genomic stability, which serves as a scaffold to promote the localization of various DDR components to DNA double-strand break (DSB) sites [100]. The deubiquitinating enzyme ubiquitin-specific protease 7 (USP7), also known as HAUSP, has been identified as an oncogene with essential roles in tumorigenesis and therapeutic resistance for a number of cancer types. Notably, overexpressed USP7 was positively correlated with the expression of MDC1 and worse survival for patients with cervical cancer. Significantly, USP7 physically interacted with and modulated the stabilization of MDC1, thereby sustaining the DDR and conferring cellular resistance to genotoxic insults [101]. Checkpoint kinase 1 (CHK1) and checkpoint kinase 2 (CHK2) are well-established signal transducers for checkpoint-mediated cell cycle arrest and activation of DNA repair in response to DNA damage or unreplicated DNA [102, 103]. USP7 interacted with, and stabilized CHK1 protein levels and functions through K48 deubiquitylation in acute myeloid leukemia (AML) [104]. Cell division cycle 25 A (CDC25A) is a highly conserved, dual-specificity phosphatase that regulates the cyclin-dependent kinases (CDKs) [105]. USP7 was also found to extend the half-life of CDC25A by circumventing turnover, which stabilized CDC25A and enhanced resistance to DNA-damaging agents in cervical cancer [106]. P53 is the most frequently mutated tumor suppressor in human cancer [107]. USP7 is known to stabilize the oncogenic E3 ubiquitin ligase mouse double minute 2 homolog (MDM2) that promotes the proteasomal degradation of p53 [108]. A recent study revealed that the USP7-degrading proteolysis targeting chimera (PROTAC) maintained potent cell growth inhibition in p53 mutant cancer cells, suggesting USP7-PROTAC as a promising method for potential p53 mutant cancer therapy [109]. Polo-like kinase 1 (PLK1) is a master mitotic regulator regulating DNA damage, the G2/M checkpoint, cell death pathways, and replication stress response [110]. USP7 also regulates spindle checkpoints by sustaining the protein stability of PLKI via the C233 site. Knockdown of either USP7 or PLK1 induces cell apoptosis and cell cycle G2/M arrest, resulting in reduced taxane resistance [111]. In summary, these data indicate that USP7 is of potential to be a marker and therapeutic target in overcoming resistance to treatment.

Except for USP7, which modulates the stability of p53, other types of DUBs also participate in controlling the ubiquitination of p53 and its subsequent degradation. For example, functional USP24 is required for p53 activation/stabilization upon DNA damage. Importantly, USP24 depletion inhibits p53 upregulated modulator of apoptosis (PUMA) activation and poly (ADP-ribose) polymerases (PARP) cleavage in response to ultraviolet (UV) damage, which renders cells resistant to apoptosis in a p53-dependent manner [112]. Similarly, USP28 can be cleaved and inactivated by caspase-8, preventing USP28 from deubiquitinating and stabilizing p53, thereby overriding the p53-dependent G2/M cell cycle checkpoint and eventually leading to treatment resistance [113]. Additionally, USP39 deubiquitinates and stabilizes CHK2 in lung cancer, and the knockdown of USP39 compromises G2/M checkpoint, thereby conferring decreased apoptosis and resistance to chemotherapy and radiotherapy [114]. Moreover, increased USP3 transcriptionally modulated by Smoothened (Smo) reduces Claspin polyubiquitination and proteasomal degradation, leading to activation of Claspin-dependent ATR-CHK1 signaling and radiation resistance of glioblastoma (GBM) [115].

Notably, E3s are implicated in the modulation of cell cycle checkpoints in response to DNA damage. For example, MDC1, an essential protein that triggers the DNA damage response, can be ubiquitinated and subsequent degraded by RNF4, which can be reversed by deubiquitinating enzyme ATX3 [116]. HUWE1 is a prominent CHK1 E3 ubiquitin ligase. HUWE1 knockdown results in stabilization of CHK1 and the regulation of the DDR signaling pathway [117]. PIRH2 and SIAH2 have also been shown to regulate the stability of CHK2, with significant consequences on cell cycle control involved in DDR [118, 119].

Additionally, E3 ubiquitin ligases regulate the expression levels and activities of p53 in response to exogenous and endogenous stresses. MDM2, a master regulator of p53, destabilizes p53 and causes cisplatin resistance in multiple cancers, including melanoma, ovarian, and lung cancer [120–122]. Moreover, microrchidia family CW-type zinc finger 2 (MORC2) is characterized as an oncogenic protein that facilitates DNA repair through a PARP-1-dependent mechanism [123]. Protein kinase cAMP-activated catalytic subunit alpha (PRKACA)-mediated phosphorylation of MORC2 on T582 abrogates CMA-mediated degradation of MORC2, leading to endocrine resistance of breast cancer cells [124].

### Double-strand break repair

DNA double-strand breaks (DSBs) are considered one of the most lethal forms of DNA damage [125]. Homologous recombination (HR) and nonhomologous end-joining (NHEJ) are two major approaches to the repair of DSBs [126]. HR repair of DSBs requires homologous DNA as a template. In this process, the tumor suppressors BRCA1, BRCA2, BARD1, and RAD51 are indispensable for HR execution [127]. In NHEJ, DSBs are first recognized by the Ku70-Ku80 heterodimer (Ku). They then recruit other NHEJ proteins to the DNA ends, including DNA-dependent protein kinase catalytic subunit (DNA-PKcs) and DNA ligase 4 (LIG4) [128]. NHEJ is the predominant pathway for the repair of DSBs in all mammalian cells [129]. However, the HR mechanism is either absent or impaired in BRCA1/2 mutated cells [130]. In this case, the PARP enzymes are activated and serve as a complementary approach for DNA repair. Thus, PARP inhibitors (PARPi) are effective targeting agents by competitively inhibiting the activity of PARP in cancers with BRCA1/BRCA2 mutation [131].

Remarkably, several regulators have been shown to promote drug resistance by targeting homologous recombination. For example, HERC2 has been identified as the specific E3 ligase of BRCA1 for degradation [132]. By contrast, abnormal spindle-like microcephaly-associated protein (ASPM) can promote homologous recombination by safeguarding BRCA1 stability by preventing its E3 ligase HERC2 from accessing BRCA1 under UV-induced DNA damage [133]. Correspondingly, the deubiquitinating enzyme USP9X stabilizes BRCA1 from degradation and promotes resistance to DNA-damaging agents in various types of cancer cells [134]. c-Jun N-terminal kinase 1/2 (JNK1/2)-mediates PARP-1 phosphorylation and subsequent degradation, while mitogen-activated protein kinase phosphatase-1 (MKP-1) inhibits the activity of JNK1/2 to suppress PARP-1 degradation in cisplatin-resistant ovarian cancer [135]. However, the E3 ligase of PARP-1 and the regulatory mechanism still need further investigation. Consistently, DOC-2/DAB2 interactive protein (DAB2IP) can form a complex with PARP-1 and its identified E3 ligase in renal cell carcinoma (e.g., RanBP2, TRIP12, RNF40, and VHL), leading to PARP-1 degradation. DAB2IP deficiency facilitates ionizing radiation resistance [136]. In addition to BRCA1 and PARP, several other proteins also participate in the regulation. For instance, Kelch-like ECH-associated protein 1 (KEAP1) mediates EMSY degradation, leading to enhanced homologous recombination and PARPi resistance in lung cancer [137]. In addition, exonuclease 1 (EXO1) is responsible for 3' ssDNA formation of DSB end resection, whose degradation mediated by RING-box protein 1 (RBX1) attenuates the homologous recombination pathway [138]. The RNA methyltransferase TRDMT1 has been newly identified to facilitate homologous recombination, the degradation of which by TRIM28-mediated ubiquitination results in sensitizing ovarian cancer to platinum treatment [139]. In addition to homologous combination, several E3 ligases participate in the regulation of NHEJ. RNF144A induces degradation of cytoplasmic DNA-PKcs, which is attributed to impaired NHEJ and increased sensitivity to DNA-damaging agents [140]. FBXW7 facilitates NHEJ in a degradation-independent manner via K63-linked polyubiquitination of X-ray repair cross-complementing protein 4 (XRCC4) at lysine 286, implying that inactivating FBXW7 could be a potential strategy for improving the efficacy of radiotherapy in human cancers [141].

### Mismatch repair

DNA mismatch repair (MMR) is an evolutionarily conserved process that corrects base–base mismatches and insertion/deletion generated during DNA replication and recombination [142, 143]. MutS protein homolog 2 (MSH2) is the main component of the MMR system, which binds to DNA mismatches, thereby triggering cell cycle arrest or apoptosis [144]. Recent advances provide insight into the protein level of MSH2 modulated by DUBs or E3s, as well as its profound influence on MMR and treatment tolerance [145]. Previous studies have shown that HDAC6 has E3 ligase activity to promote the degradation of MSH2, leading to reduced cellular sensitivity to DNA-damaging agents [146]. In addition, DUBs have also been found to regulate MSH2. Notably, USP10 stabilizes MSH2 and reverses resistance to DNA-methylating agents and antimetabolite 6-thioguanine (6-TG) in lung cancer cells [147]. Likewise, the association and clinical implication of USP10 and MSH2 proteins are further confirmed in NSCLC tissues, indicating that USP10 stabilizes MSH2 in patients with lung cancer [148].

Taken together, a wide range of DUBs and E3s play crucial roles in regulating DNA repair by regulating DDR, cell cycle checkpoints, DSB repair, and MMR. DUB or E3 inhibitors are potential therapeutic agents for overcoming drug resistance.

## Regulation of targeted therapy through modulating protein degradation

Drug efficacy of targeted therapy is largely determined by alterations of the drug targets, such as protein levels [149, 150]. Alteration of common oncogenic proteins, such as epidermal growth factor receptor (EGFR), KRAS, BRAF, and c-Myc, drives tumorigenesis across 38 cancer types [151, 152]. Multiple evidence shows that several DUBs and E3s are correlated with resistance to targeted therapy.

### EGFR

EGFR (also known as ERBB1 or HER1) is a member of the ERBB family of cell surface receptor tyrosine kinases [153]. The binding of ligands triggers receptor dimerization, tyrosine phosphorylation, and activation of downstream signaling pathways, including RAS-mitogen‑activated protein kinase (MAPK), phosphoinositide 3-kinase (PI3K)-AKT, and Janus kinase (JAK)-signal transducer and activator of transcription (STAT), leading to cell proliferation [154]. Following interaction with ligands, EGFR is activated and subsequently internalized into endosomes, where it can be either recycled to the membrane surface or degraded through K63-ubiquitin chain-mediated lysosomal degradation [155]. In addition, the proteasomal degradation pathway also participates in the degradation control of EGFR [156]. Despite numerous EGFR tyrosine kinase inhibitors (TKIs) exhibiting marked efficacy, resistance to these agents remains an unsolved fundamental challenge, where dysregulation of EGFR degradation may be one of the pivotal reasons [11].

Studies have revealed that E3s are vital novel players in the regulation of proteins implicated in EGFR-TKI resistance. For instance, casitas b-lineage lymphoma-b (Cbl-b) increased the sensitivity of gastric cancer cells to cetuximab at least partially by affecting EGFR expression [157]. Cbl-b overexpression partially reversed drug resistance to icotinib in EGFR-mutant NSCLC cells [158]. In human pancreatic cancer, low expression of Cbl conferred chemoresistance via stress-induced EGFR activation [159]. Furthermore, Cbl-b was expressed at a low level in MDR gastric and breast cancer cells compared with their parental cells [160]. A recent study provides a more detailed possible mechanism through which the membrane protein sarcoglycan epsilon (SGCE) stabilizes EGFR for breast cancer stem cell (BCSC) maintenance by disrupting the interaction between EGFR and its E3 ligase c-Cbl [161]. Likewise, the cell growth regulator with RING finger domain protein 1 (CGRRF1) functioned as a tumor suppressor and identified EGFR as its target in breast cancer [162]. Suppressor of cytokine signaling 5 (SOCS5), with E3 ubiquitin ligase activity, led to a marked reduction in EGFR expression levels by promoting ubiquitin-mediated EGFR degradation [163]. Furthermore, the combination of JAK and EGFR inhibitors overcomes acquired resistance to EGFR-TKIs since JAK2 inhibition uncouples the role of SOCS5 in the negative regulation of EGFR [164]. Additionally, RNF126, Rabring7, breast cancer-associated gene 2 (BCA2), and ZNRF1 are involved in the endocytosis of EGFR through their E3 ligase activity [165–167]. In-depth studies on E3-mediated alterations in EGFR may deepen our understanding of the development of potential strategies to reverse EGFR-TKI tolerance.

Apart from the downregulation of E3 ligases, DUBs also play an essential role in EGFR alteration. STAMBP (also known as AMSH) is a deubiquitinating enzyme of the Jab/MPN metalloenzyme family. High STAMBP levels predict poor survival in lung adenocarcinoma (LUAD) patients. Importantly, increased STAMBP promoted the stabilization of EGFR, suggesting STAMBP as a novel target for LUAD therapy [168]. Ubiquitin-specific protease 22 (USP22) has been shown to prevent ubiquitination-mediated EGFR degradation and activate multiple EGFR downstream signaling pathways, thus conferring resistance to EGFR-TKIs in mutant lung adenocarcinoma cells [169]. Moreover, ubiquitin carboxy terminal hydrolase-L1 (UCHL1) is involved in regulating the degradation of EGFR and promoting malignant properties in drug-resistant breast cancer, where the specific molecular mechanism needs further research [170]. Another independent study revealed that OTU domain-containing protein 7B (OTUD7B) served as a downstream gene of linc00976, deubiquitinated EGFR, and activated the MAPK signaling pathway. Thus, the linc00976/miR-137/OTUD7B/EGFR axis may act as a potential therapeutic target for pancreatic cancer [171]. Intriguingly, USP13 stabilizes mutant EGFR in a peptidase-independent manner by interacting with the EGFR-Cbl-b complex to protect EGFR from lysosomal degradation [172]. Hence, the combination of USP13 inhibitor spautin-1 with EGFR inhibitors shows a potent antitumor effect in vivo [173]. Overall, DUBs are intimately related to modulating the stability of EGFR, thus conferring resistance to EGFR-TKIs in cancer cells. Targeting these DUBs may present a promising strategy to reverse therapeutic resistance and achieve better clinical outcomes for EFGR-mutant patients.

### KRAS

Kirsten rat sarcoma (KRAS) is a small membrane-bound GTPase (guanosine triphosphate hydrolase) that primarily binds to guanosine diphosphate (GDP) and is inactive [174]. Upon GDP to GTP exchange, usually in response to growth factors, KRAS cycles to an activated state, allowing multiple downstream signaling pathways to be activated, including the MAPK and PI3K pathways [175]. KRAS mutations are among the most common drivers of human cancer and are associated with tumorigenesis and poor prognosis [176]. Over the past years, despite inhibitors having shown remarkable clinical responses in KRAS-mutant cancers, resistance to KRAS inhibitors has eventually developed [177]. Thus, further understanding the mechanisms of drug resistance to KRAS-mutant inhibitors is indispensable. E3 ligase *β*-transducin repeat-containing protein (*β*-TrCP) facilitates simultaneous ubiquitination and degradation of KRAS, and intriguingly, the stability of *β*-TrCP is regulated by a critical E3:E2 complex composed of Smad ubiquitination regulatory factor 2 (SMURF2) and UBCH5 [178]. In addition, E3 ligases anaphase-promoting complex subunit 2 (ANAPC2) promotes KRAS degradation through the ubiquitin–proteasome pathway in colorectal cancer [179]. Leucine zipper-like transcriptional regulator 1 (LZTR1) interacts with the CUL3-based E3 ubiquitin ligase complex, which is reported to participate in KRAS ubiquitination [180]. Mechanistically, LZTR1 ubiquitinates KRAS with K48 and K63 chains, indicating that the degradation of KRAS depends on both proteasomal and lysosomal degradation, with proteasomal degradation playing a dominant role [181]. Taken together, the identification of E3s affords fascinating potential targets to reverse drug target-associated resistance owing to their biological activity and druggability.

### BRAF

BRAF, a member of the RAF gene family, encodes a serine–threonine protein kinase that is an upstream factor of the MAPK pathway [182, 183]. Frequent mutations of BRAF (most commonly the V600E mutation) substantially increase kinase activity to drive the proliferation of cancer cells [184]. Therefore, several BRAF inhibitors have been developed and evaluated clinically for BRAF-mutated patients [185, 186]. Unfortunately, the emergence of acquired resistance to BRAF inhibitors (BRAFi) was inevitable and is one of the main reasons for therapy failure in BRAF-mutant cancers.

FBXW7 (also known as CDC4) is a component of the S-phase kinase-associated protein 1 (SKP1)/CUL1/F-box (SCF) E3 ubiquitin ligase complex, which functions as a tumor suppressor which is frequently altered in cancer [187, 188]. It has been reported that loss of FBXW7 in the presence of the BRAF^V600E^ mutation is consequential and sufficient to drive tumorigenesis in mouse models [189]. Additionally, a high-throughput reversed-phase protein array revealed BRAF as a novel target of FBXW7 in adult T cell leukemia cells. Further experiments showed that mutations in FBXW7 prevent the degradation of BRAF-conferred resistance to bromodomain and extra-terminal (BET) inhibitors [190]. Intriguingly, it has been reported that loss of the deubiquitinating enzyme USP28 can destabilize its substrate FBXW7, leading to BRAF stabilization and eventually resistance to BRAF inhibitor therapy in both in vitro and in vivo models [191]. In addition, another E3 ligase, RNF149 was identified as a wild-type BRAF-interacting protein, which induced ubiquitination of wild-type BRAF and promoted its proteasomal degradation. However, RNF149 cannot bind to mutant BRAF, which may partially explain the abnormal activation of BRAF downstream pathways in BRAF-mutant patients [192].

Increasing evidence indicates that DUBs play essential roles in acquired resistance to BRAF inhibitors. USP14 participates in the resistance to BRAF inhibitors in melanoma cells. At the molecular level, targeting USP14 in melanoma increases oxidative and proteotoxic stress and subsequently triggers ROS-dependent and caspase-independent cell death that overcomes resistance to BRAF inhibitors [193]. Moreover, USP5 deprivation reversed resistance to vemurafenib and sensitized BRAF-mutant melanoma cells to apoptosis initiated by BRAF inhibitors. Thus, targeting USP5 offers a potential therapeutic strategy for BRAF inhibitor-resistant melanoma [194]. Together, unveiling the determinants of DUB and E3 functions in modulating acquired resistance to BRAFi may facilitate the development of new strategies to sensitize cancer cells to BRAFi.

### c-Myc

c-Myc, a pleiotropic transcription factor, is considered one of the essential drivers in various types of tumors by controlling global gene expression including cell cycle, proliferation, differentiation, metabolism, and apoptosis [195]. Although it has excellent potential as a therapeutic target, c-Myc has long been considered untargetable due to its protein structure [196]. Recent studies revealed that E3 ligase MAGI3 promotes ubiquitination and degradation of c-Myc in colorectal cancer cells, whose expression can act as a predictor for chemotherapy response and patient survival [197]. Similarly, E3 ligase FBW7*α* inhibits the proliferation and progress of cholangiocarcinoma and hepatocellular carcinoma by downregulating c-Myc [198, 199]. In addition, deubiquitinating enzymes USP22 [200], USP28 [201], and OTUD6A [202] stabilize c-Myc and promote tumorigenesis in breast cancer, squamous cell lung carcinoma, and prostate cancer, respectively. Hence, regulating the expression levels of specific E3s and DUBs may provide a more convenient and effective clinical treatment method for targeting c-Myc.

## Downstream adaptive responses mediated by protein degradation

**Fig. 5:**
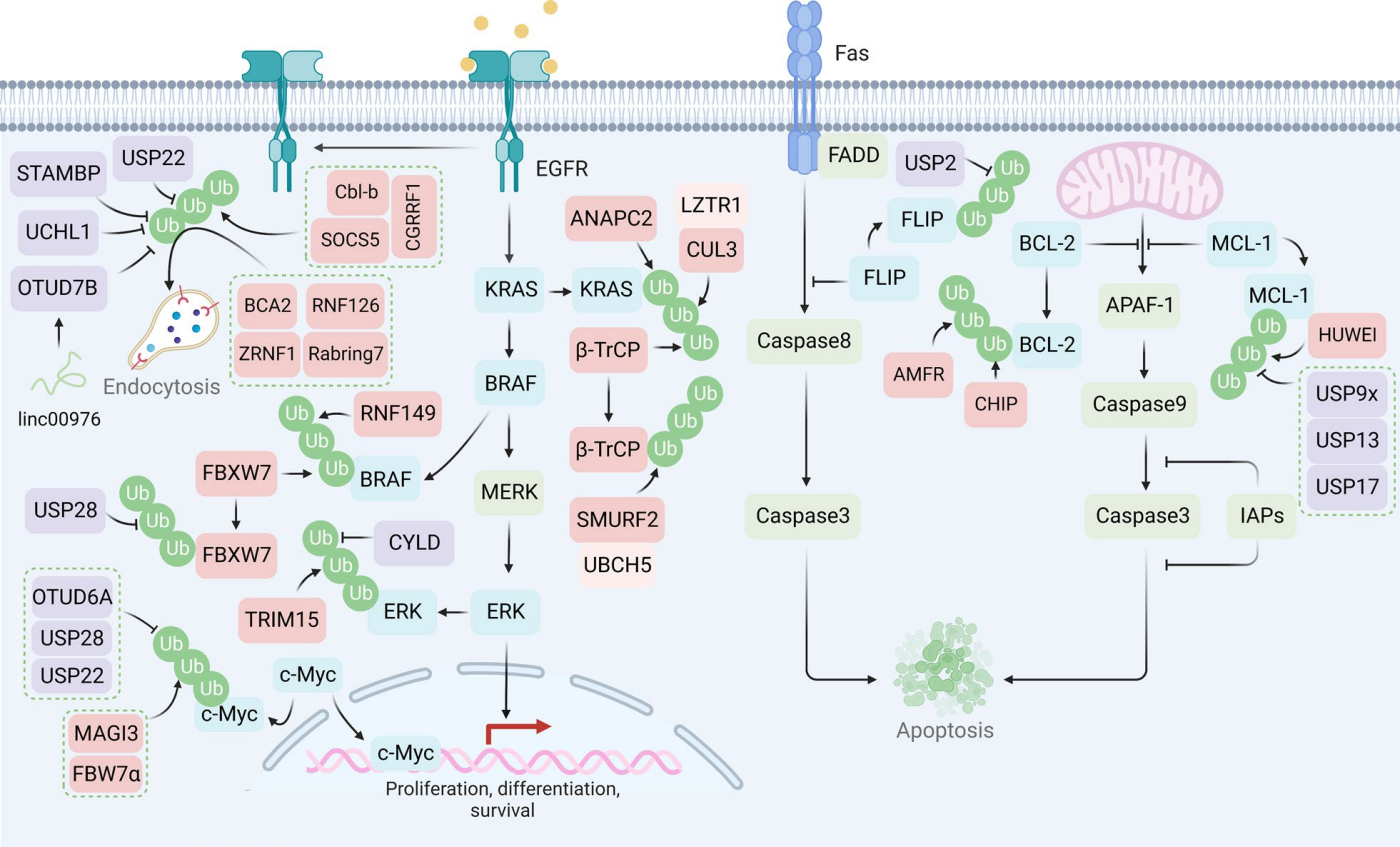
Protein degradation regulates the efficacy of targeted therapy and chemotherapy. SOCS5, CGRRF1, and Cbl-b promote ubiquitination and subsequent endocytosis of the EGFR, while UPS22, STAMBP, UCHL1, and OTUD7B deubiquitinate EGFR. E3 ligases FBXW7 and RNF149 facilitate ubiquitination of BRAF. USP28 regulates the stability of BRAF by deubiquitinating FBXW7. ANAPC2, *β*-TrCP, and LZTR1/CUL3 complexes promote KRAS ubiquitination for degradation. E3 ligase TRIM15 and CYLD regulate the ubiquitination and deubiquitylation of ERK, respectively. E3 ligase MAGI3 and FBW7*α* facilitate the ubiquitination of c-Myc, and USP22, USP28, and OTUD6A stabilize c-Myc. Anti-apoptosis protein FLIP can be stabilized through USP2-mediated deubiquitylation. BCL-2 is ubiquitinated by AMFR and CHIP. MCL-1 can be ubiquitinated by HUWEI and deubiquitylated by USP9X, USP13, or USP17

When therapeutic drugs successfully bind to the effectors, pro-death signaling pathways are usually activated (chemotherapy) or pro-survival signaling pathways inhibited (targeted therapy) [203]. However, during tumor treatment, drug-resistant tumor cells are often able to evade cell death by upregulating anti-apoptotic proteins and reactivating pro-survival signaling pathways through the bypass mechanisms (Fig. 5).

### Evasion of apoptosis

Apoptosis is one of the important mechanisms for first-line chemotherapeutic drugs (e.g., cisplatin, paclitaxel) in tumor therapy and is triggered by drug-mediated DNA damage, cellular stress, and immune response [204]. Selective protein degradation may promote drug resistance through the downregulation of pro-apoptotic proteins and upregulation of anti-apoptotic proteins, including B cell lymphoma 2 (BCL-2) and FLICE-inhibitory protein (FLIP). For example, USP17 stabilizes MCL-1, a BCL-2 family protein, through deubiquitylation, leading to chemoresistance in ovarian cancer [205]. Consistently, USP9x and USP13 also promote drug resistance by stabilizing MCL-1 [206, 207]. USP9x is also found to deubiquitinate and stabilize pre-B-cell leukemia homeobox-1 (PBX1) in advanced prostate cancer (PCa), thereby abrogating apoptosis and conferring chemoresistance to doxorubicin or cisplatin [208]. In addition, USP2 promotes sorafenib resistance by deubiquitinating cFLIP in hepatocellular carcinoma [209]. However, some DUBs may induce drug resistance by regulating pro-apoptotic proteins. For instance, downregulated USP15 leads to imatinib resistance by regulating the degradation of caspase-6 in myeloid leukemia cells [210], whereas blocking the DUB activity of Rpn11 activates caspase cascade and triggers apoptosis, providing the rationale for overcoming bortezomib resistance in multiple myeloma (MM) [211].

Apart from DUBs, E3 ligases also regulate the degradation of apoptosis-related proteins. The expression of the E3 ligase AMFR has been validated to be negatively correlated with the expression of the anti-apoptotic protein BCL-2 [212]. CHIP is a chaperone-dependent and U-box-containing E3 ligase that negatively correlates with breast cancer clinicopathological stages and overall survival [213]. CHIP acts as a capacitor of heterogeneous BCL-2 expression levels and prevents an increase in the anticancer drug-resistant population in breast cancer cells [214]. Moreover, cullin-RING ubiquitin ligases 4 (CRL4) E3 ligase has been reported to be upregulated and is associated with poor prognosis in ovarian cancer patients. Mechanistically, CRL4A-DDB1 stimulates STAT3 activation by degrading STAT1, leading to increased expression of Baculoviral IAP repeat containing 3 (BIRC3), one of the inhibitors of apoptosis proteins (IAPs), eventually resulting in chemoresistance against cisplatin [215]. In the intrinsic apoptotic pathway, intracellular apoptotic stimulations promote the release of cytochrome c from mitochondria, triggering activation of apoptotic protease activating factor 1 (APAF-1) and eventually activating caspase-9 apoptosome [216]. In breast cancer cells that acquired resistance to lapatinib, MDM2 ubiquitinates another ubiquitin E3 ligase, HUWE1, which indirectly inhibits apoptosome activation by preventing HUWE1 from ubiquitinating MCL-1 [217]. Furthermore, high-throughput screening revealed that RING finger containing E3 ligase SIAH2 and the signaling platform molecule SH3 domain containing RING finger 1 (SH3RF1) confer robust caspase-8 activation and suggest targeting the interaction of these two E3 ligases is a promising novel cancer therapeutic strategy [218].

Intriguingly, chaperone-mediated autophagy is reported to participate in the regulation of apoptosis. Inhibition of CMA through LAMP2A knockdown increases the expression of p53 and Bax, sensitizing the cisplatin resistance of lung cancer cells [219].

### Oncogenic bypass signaling

Although targeted therapy has been developed to block specific signaling pathways in tumor progression, drug resistance still occurs due to the activation of oncogenic bypass signaling pathways (e.g., the MAPK pathway) [176, 220]. The MAPK pathway is one of the most important signaling pathways involved in the reactivation of downstream oncogenic transfactors [221]. As a USP class of cysteine proteases, cylindromatosis-associated DUB (CYLD) is identified as a tumor suppressor because its expression is inversely correlated with overall and progression-free survival. Of interest, CYLD and the E3 ligase of the extracellular signal-regulated kinase (ERK), tripartite motif-containing protein 15 (TRIM15), have been shown to participate in regulating ERK activation via the modulation of its lysine-63-linked polyubiquitination, which is important for the survival of therapeutic-resistant melanoma [222]. Cytokines activate ITCH to maintain BRAF activity and promote the tumorigenicity of melanoma cells. Mechanistically, ITCH-mediated lysine 27-linked ubiquitination of BRAF recruits protein phosphatase 2A (PP2A) to antagonize S365 phosphorylation and disrupt the inhibitory interaction with 14–3–3, leading to sustained activation of BRAF downstream MEK/ERK signaling [223]. However, whether cytokines confer resistance to BRAF inhibitors in such an ITCH-dependent manner needs further elucidation.

Recently, overexpression of yes-associated protein (YAP) was found to be associated with resistance to BRAF inhibitors in melanoma. A recent study demonstrated that ubiquitin-specific peptidase 22 (USP22) interacted with and stabilized YAP, which in turn conferred resistance to the BRAF inhibitor vemurafenib and provided new therapeutic avenues to target USP22/YAP as an option for melanoma treatment [224]. RNF44 was proposed to promote AMP-activated protein kinase *α*1 (AMPK-*α*1) degradation and consequently regulate autophagy and metabolic reprogramming in BRAFi-resistant melanoma cells [225]. Microtubule-associated serine/threonine kinase 1 (MAST1) is the main driver of drug resistance through rewiring RAF-independent MEK activation [226]. The E3 ligase CHIP can promote MAST1 degradation by ubiquitination on Lys 317 and Lys 545, which can be abrogated by HSP90B-mediated protection of ubiquitinylating sites [227]. Therefore, targeting HSP90B can predominantly sensitize cancer cells to cisplatin through MAST1 destabilization.

## Cancer cell stemness and protein degradation

**Fig. 6:**
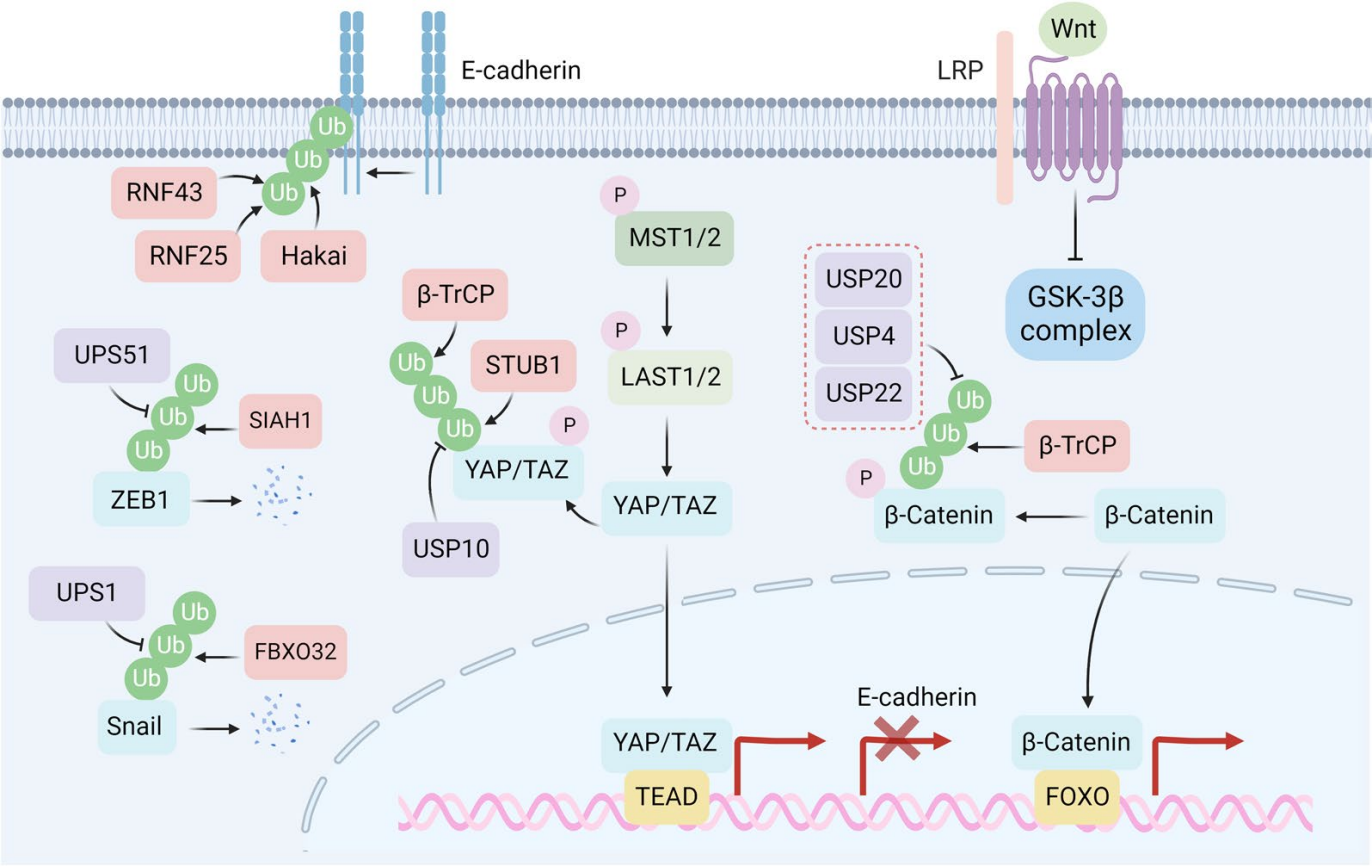
Protein degradation involved in EMT and cell stemness. Degradation of E-cadherin is regulated by E3 ligases RNF25, RNF43, and Hakai. Protein degradation of mesenchymal marker ZEB1 is mediated by E3 ligase SIAH1 and can be reversed by USP51. Snail is ubiquitinated by FBXO32 and deubiquitinated by USP1. For stemness sustaining pathways, *β*-TrCP participates in the ubiquitination of both YAP and *β*-catenin. In addition, STUB1 also facilitates the ubiquitination of YAP. USP10 facilitates deubiquitylation of YAP, and USP20, USP4, and USP22 deubiquitinate *β*-catenin

Cancer stem cells (CSCs) are a small subpopulation of tumor cells capable of self-renewal, enabling drug tolerance [228]. CSCs can be initialized through epithelial-to-mesenchymal transition (EMT) [229] and sustained by stemness-related pathways (e.g., the Notch pathway, Wnt pathway, and Hedgehog and Hippo pathways) [230]. Recent studies indicate that the molecular mechanisms of protein degradation regulate EMT- and stemness-related pathways (Fig. 6).

Cancer cells that undergo the EMT process may gain stem-like properties and a high potential for metastasis, leading to treatment refractoriness [231–233]. Loss of E-cadherin, the essential component of cell-to-cell conjugation, is considered a fundamental event in the EMT process [234, 235]. The E3 ligases Hakai and RNF43 have been reported to participate in the ubiquitin-related degradation of E-cadherin, facilitating the EMT process in tumor progression [236, 237]. Intriguingly, a recent study reveals that RNF25 plays an essential role in oxidative stress-mediated E-cadherin degradation during liver cancer metastasis [238], suggesting that changes in the tumor microenvironment may also affect the cellular protein degradation process. Apart from direct regulation of E-cadherin protein degradation, several EMT-inducing transcription factors (e.g., ZEB1, Snail, and Twist) can downregulate E-cadherin expression by repressing its transcription [239]. Downregulation of the E3 ligase SIAH1 stabilizes ZEB1 in doxorubicin-resistant HCC cells [240]. Consistently, USP51 deubiquitinates ZEB1 resulting in a poor prognosis in breast cancer patients [241]. Dysregulation of FBXO32, an E3 ligase of Snail, leads to elevated Snail expression and acquired platinum resistance in urothelial carcinoma [242]. USP1 eliminates the polyubiquitin chain on Snail, leading to resistance to platinum in ovarian cancer [243]. Conversely, RNF8 induces K63 ubiquitination of Twist to promote its translocation to the nucleus for subsequent pro-EMT and CSC functions, leading to cancer chemoresistance [244]. The context-dependent functions of ubiquitination modification suggest that the clinical application of targeted ubiquitination must consider different application scenarios.

The maintenance of CSCs is closely related to the activation of embryonic developmental pathways (e.g., the Wnt and Hippo pathways) [230, 245]. In the absence of Wnt ligand, *β*-catenin may be phosphorylated by the *β*-catenin destruction complex composed of Axin, glycogen synthase kinase 3*β* (GSK3*β*), casein kinase 1*α* (CK1*α*), and adenomatous polyposis coli (APC) and subsequently degraded by the E3 ligase *β*-TrCP [246]. In addition, *β*-TrCP can facilitate the degradation of YAP [247]. Hence, activating *β*-TrCP through aspirin treatment can attenuate the expression of *β*-catenin and YAP, resulting in the reversal of vinorelbine and docetaxel resistance in triple-negative breast cancer [248]. USP20 and USP4 induce drug resistance in breast cancer and colorectal cancer cells by stabilizing *β*-catenin, the key modulator of the Wnt pathway [249, 250]. Neutral red, a selective inhibitor of USP4, has shown a significant effect on suppressing colorectal cancer progression [251]. In addition, USP22 is reported to participate in colorectal cancer stemness and chemoresistance through the regulation of the Wnt/*β*-catenin pathway [252, 253]. However, whether this regulation is protein degradation-dependent needs further validation [254]. Apart from the Wnt pathway, the E3 ligase STIP1 homology and U-Box-containing protein 1 (STUB1) induce YAP degradation in gastric cancer cells, leading to cellular chemosensitivity [255]. Deubiquitylation of YAP by USP10 mediates the aberrant activation of the Hippo pathway and progression of hepatocellular carcinoma [256].

## Tumor microenvironment remodeling by protein degradation-regulated pathways

**Fig. 7:**
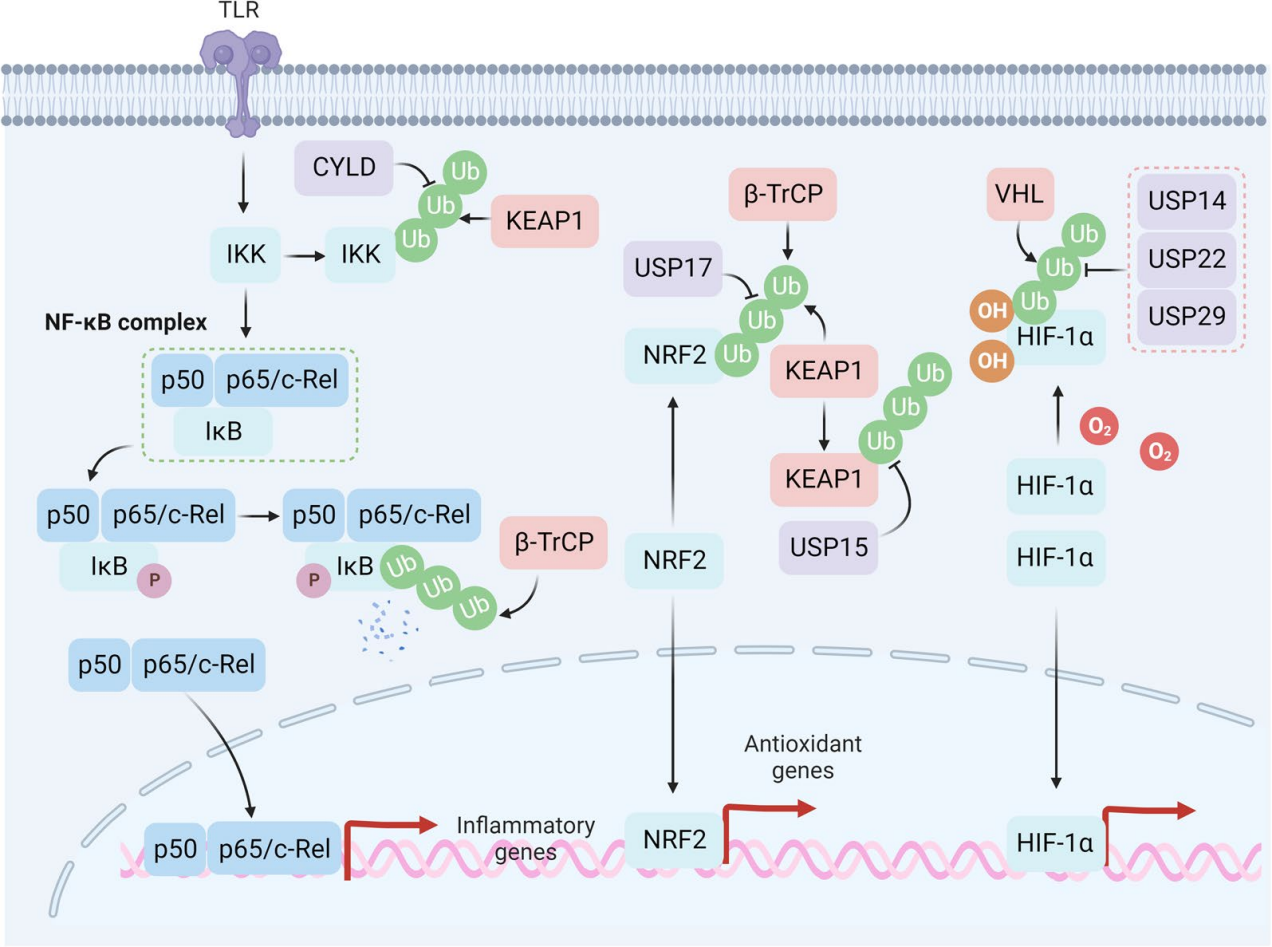
Protein degradation modulates the tumor microenvironment. IKK can be stabilized by CYLD to facilitate the phosphorylation of I*κ*B, which can be reversed by KEAP1-mediated ubiquitylation on IKK. *β*-TrCP induces activation of the NF-*κ*B pathway by ubiquitinating I*κ*B, resulting in nucleus translocation of the NF-*κ*B complex. *β*-TrCP and KEAP1 facilitate NRF2 degradation, while USP17 can deubiquitinate NRF2. KEAP1 can also be regulated by DUB USP15. Under hypoxic conditions, HIF-1*α* can translocate into the nucleus and facilitate transcription of hypoxic genes. While under normoxia, HIF-1*α* can be oxidated and further ubiquitinated by VHL for degradation. USP22, USP29, and USP14 can deubiquitylate HIF-1*α*

Besides its role in intracellular molecular mechanisms of drug resistance, the deregulation of the extracellular tumor microenvironment has attracted increasing research interest [257–259]. Recent studies indicate that tumor progression needs to overcome extrinsic stress (e.g., oxidative stress, hypoxia) caused by therapy and nutrition deprivation [260–264] (Fig. 7). Redox regulates cancer drug tolerance and dormancy, which are closely related to poor prognosis [265, 266]. Depletion of the E3 ligase FBXW7 sensitizes dormant cancer cells to paclitaxel, indicating that protein degradation may participate in the regulation of dormancy [267]. Acquired drug-resistant tumor cells, on the one hand, increase reactive oxygen species (ROS) signaling (e.g., the NF-*κ*B pathway) to maintain their active proliferative state and simultaneously activate the antioxidant defense system (e.g., NRF2, GSH, high-abundance redox proteins) to avoid oxidative stress-mediated cell death [268]. Under a normal redox state, the essential E3 ligase KEAP1 targets NRF2 for proteasome-dependent degradation. Under oxidative stress, KEAP1 may be inactivated by redox modification of Cys151, Cys273, and Cys288, resulting in the activation of NRF2-mediated transcription of antioxidant genes [269]. DUB USP17 eliminates the K48 ubiquitin chain on NRF2, resulting in camptothecin and paclitaxel resistance in colorectal cancer [270]. In addition, USP15 stabilizes KEAP1 and promotes the degradation of NRF2, leading to the sensitization of cancer cells to chemotherapy [271, 272]. Apart from KEAP1, another E3 ligase, *β*-TrCP also participates in the degradation of NRF2, whose downregulation is closely related to drug resistance in various types of cancer [273, 274]. Recent studies have reported that cyclophilin A (CYPA), a high-abundance redox protein, can buffer excessive ROS through self-oxidation on Cys115 and Cys161, leading to resistance to 5-fluorouracil and oxaliplatin in colorectal cancer [275]. Consistently, deubiquitylation of CYPA by USP4 promotes a poor prognosis of hepatocellular carcinoma [276]. Apart from the upregulation of the antioxidant defense system, CYLD-mediated deubiquitylation of inhibitor of NF-*κ*B kinase (IKK) leads to the inactivation of the NF-*κ*B pathway and reversion of drug resistance [277]. Intriguingly, KEAP1 regulates the activation of NF-*κ*B pathway through ubiquitination and degradation of IKK*β* [278]. Furthermore, *β*-TrCP is reported to participate in the ubiquitin-dependent degradation of I*κ*B [279]. In addition, the secretion of pro-inflammatory or anti-inflammatory cytokines may influence the sensitivity of anticancer agents, which may be context-dependent [280]. For instance, hepatitis B virus X-interacting protein (HBXIP) inhibits CMA-mediated homeobox B13 (HOXB13) degradation by enhancing acetylation at Lys 277, leading to the expression of pro-inflammatory cytokine IL-6 and tamoxifen resistance in breast cancer [281].

Activation of the hypoxia-inducible factor 1 alpha (HIF-1*α*) pathway under hypoxia is closely related to the transcription of drug resistance-related genes in multiple tumors [282–284]. Under a normal redox state, HIF-1*α* can be oxidized on Pro402 and Pro564 and subsequently degraded by von Hippel–Lindau protein (pVHL)-dependent degradation [285]. DUBs, including USP14, USP22, and USP29, mediate the deubiquitylation and stabilization of HIF-1*α*, promoting self-renewal ability and drug resistance in hepatocellular carcinoma [286–288]. Knockdown of CHIP was associated with high levels of HIF-1*α* and greatly enhanced growth in PCa tumor xenografts, suggesting that the use of mitotic kinase inhibitors will open new approaches for the treatment of hypoxic PCa tumors [289].

## Targeting protein degradation to surmount drug resistance

**Fig. 8:**
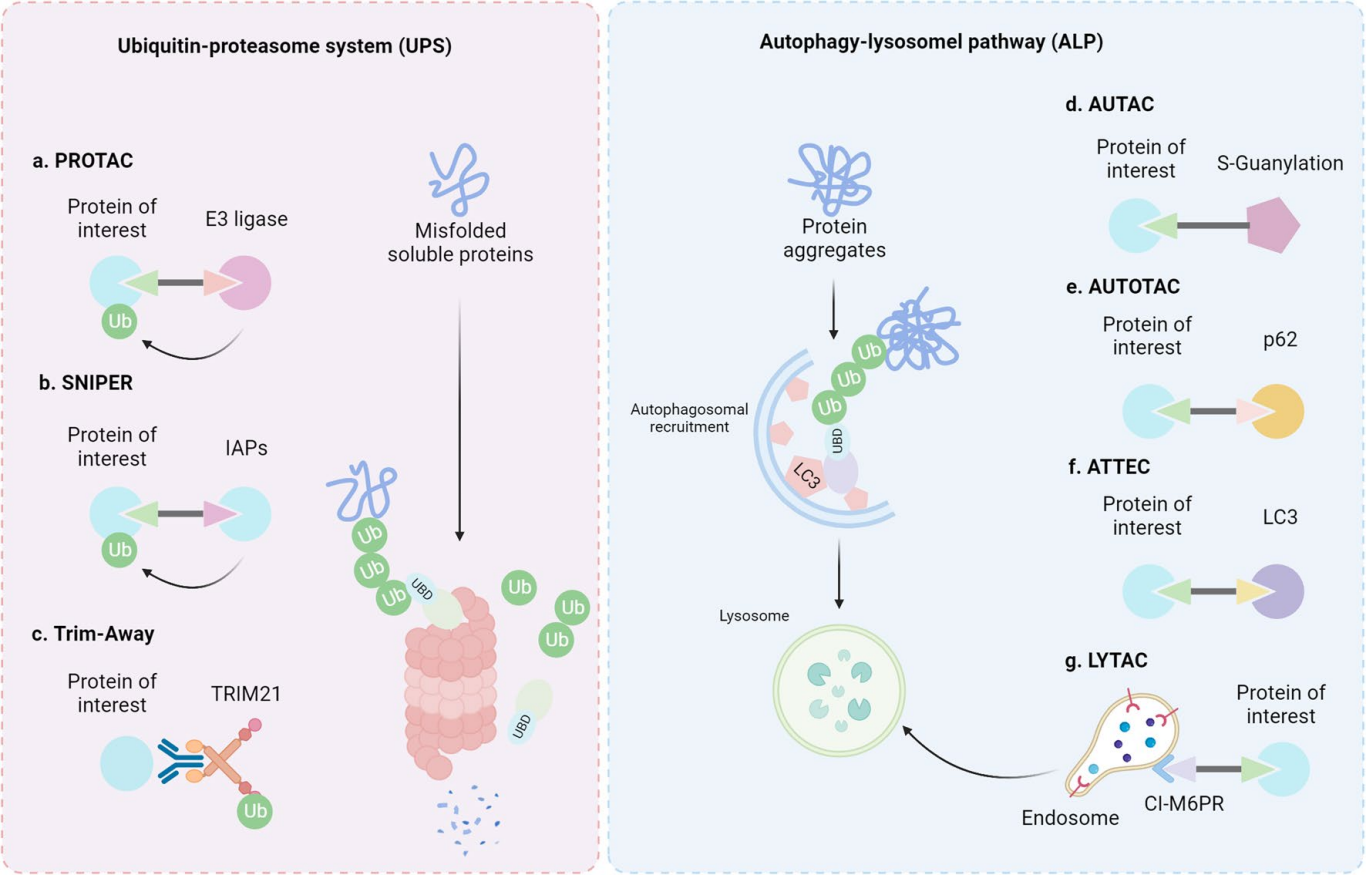
Current technologies targeting protein degradation. **a** PROTAC is designed to conjugate specific E3 ligases and proteins of interest for further degradation. **b** Based on the E3 ligase activity of IAPs, SNIPERs conjugate IAPs and proteins of interest for degradation. **c** TRIM21 has high affinity for antibodies. Therefore, using specific antibodies against the proteins of interest can lead to rapid degradation. **d** AUTAC is a heterobifunctional compound with a targeting warhead and a tag that recruits the autophagy system, which may lead to ubiquitin-dependent selective degradation through lysosomal degradation. **e** AUTOTAC connects the protein of interest and p62 for autophagy-induced protein degradation. **f** ATTEC conjugates the protein of interest and LC3 for degradation. **g** LYTAC interacts with membrane proteins or extracellular proteins and the CI-M6PR for endocytosis

Studying the regulatory mechanism of protein degradation can provide a basis for finding rapid and effective therapeutic strategies (Table 1). Several small molecules targeting E3 ligases or DUBs have entered clinical trials for treating various types of refractory tumors (Table 2). Due to the limited substrates that have been studied based on the selective regulation of E3, DUBs, and chaperones, there are quantities of proteins that are considered untargetable by modulating protein degradation [290]. Encouragingly, as a result of the in-depth study of biological mechanisms and the development of chemical synthesis technology, it has become possible to reverse the “untargetable” into a “targetable” situation [291]. At present, a variety of new technologies have been developed based on the proteasome and lysosomal pathways, which are expected to be effective in overcoming tumor drug resistance (Fig. 8).

**Table 1:** Representative regulators of targeted protein degradation involved in drug resistance

Protein degradation regulator	Category	Substrate	Therapeutic agents	Role	Tumor	Mechanism	Refs.
FBXO15	E3	P-gp	Vincristine and doxorubicin	Sensitive	CRC	Drug efflux	[54]
FBXO21	E3	P-gp	Valinomycin	Sensitive	Ovarian cancer	Drug efflux	[57]
MARCH8	E3	P-gp	Adriamycin, paclitaxel, and colchicine	Sensitive	Breast cancer, NSCLC	Drug efflux	[60]
AMFR	E3	P450s	Enzalutamide	Resistant	–	Drug metabolism	[77]
FBXW7	E3	BRAF	BET inhibitors	Sensitive	T cell leukemia	Altering drug target	[190]
Cbl-b	E3	EGFR	Adriamycin	Resistant	BC/GC	Altering drug target	[160]
SIAH2	E3	CHK2	Genotoxic agents	Sensitive	Multiple cancer types	DNA repair	[118]
MDM2	E3	p53	Cisplatin	Resistant	Uveal melanoma, ovarian cancer, lung cancer	DNA repair	[120–122]
TRIM28	E3	TRDMT1	DNA-damaging agents	Sensitive	Ovarian cancer	DNA repair	[139]
RNF144A	E3	DNA-PKcs	DNA-damaging agents	Sensitive	Multiple cancer types	DNA repair	[140]
CHIP	E3	BCL-2	Chemotherapy	Sensitive	Breast cancer	Apoptosis	[214]
CRL4	E3	STAT1	Cisplatin	Resistant	Ovarian cancer	Apoptosis	[215]
RNF44	E3	AMPK-*α*1	BRAFi	Resistant	Melanoma	Oncogenic bypass signaling	[225]
CHIP	E3	MAST1	Cisplatin	Sensitive	Multiple cancer types	Oncogenic bypass signaling	[227]
SIAH1	E3	ZEB1	Doxorubicin	Sensitive	HCC	EMT	[240]
FBXO32	E3	Snail	Platinum	Sensitive	Urothelial carcinoma	EMT	[242]
*β*-TrCP	E3	*β*-catenin and YAP	Vinorelbine and docetaxel	Sensitive	Triple-negative breast cancer	Stemness	[248]
*β*-TrCP	E3	NRF2	Temozolomide	Sensitive	Glioma	ROS signaling	[273]
*β*-TrCP	E3	NRF2	Chemotherapy	Sensitive	Pancreatic cancer	ROS signaling	[274]
USP28	DUB	BRAF	BRAF inhibitors	Resistant	Melanoma	Altering drug target	[191]
USP14	DUB	BRAF	BRAF inhibitors	Resistant	Melanoma	Altering drug target	[193]
USP22	DUB	EGFR	EGFR-TKIs	Resistant	Lung adenocarcinoma		[169]
USP17	DUB	EGFR	EGFR-TKIs	Resistant	NSCLC	Altering drug target	[292]
UCHL1	DUB	EGFR	EGFR-TKIs	Resistant	Breast cancer	Altering drug target	[170]
USP7	DUB	MDC1	Genotoxic insults	Resistant	Cervical cancer	DNA repair	[101]
USP7	DUB	CHK1	Cytarabine	Resistant	Acute myeloid leukemia	DNA repair	[104]
USP7	DUB	CDC25A	DNA-damaging agents	Resistant	Cervical cancer	DNA repair	[106]
USP7	DUB	PLK1	Taxane	Resistant	Lung cancer	DNA repair	[111]
USP24	DUB	p53	Radiation	Sensitive		DNA repair	[112]
USP28	DUB	p53	Genotoxic chemotherapy	Sensitive	Multiple cancer types	DNA repair	[113]
USP39	DUB	CHK2	Chemotherapy drugs and radiation treatment	Sensitive	Lung cancer	DNA repair	[114]
USP3	DUB	Claspin	Radiation	Resistant	Glioblastoma	DNA repair	[115]
USP9x	DUB	BRCA1	DNA-damaging agents	Resistant	Multiple cancer types	DNA repair	[134]
USP10	DUB	MSH2	*N*-methyl-*N*′-nitro-*N*-nitrosoguanidine (MNNG) and 6-thioguanine (6-TG)	Sensitive	Lung cancer	DNA repair	[148]
USP2	DUB	cFLIP	Sorafenib	Resistant	Hepatocellular carcinoma	Apoptosis	[209]
USP17	DUB	Mcl-1	Platinum and paclitaxel	Resistant	Ovarian cancer	Apoptosis	[292]
USP9x	DUB	Mcl-1	Ionizing radiation	Resistant	Oral cancer	Apoptosis	[206]
USP15	DUB	Caspase-6	Imatinib	Sensitive	Chronic myeloid leukemia	Apoptosis	[210]
USP1	DUB	Snail	Platinum	Resistant	Ovarian cancer	EMT	[243]
USP51	DUB	ZEB1	Cisplatin	Resistant	Lung cancer	EMT	[293]
USP20	DUB	*β*-catenin	Chemotherapy	Resistant	Breast cancer	Stemness	[249]
USP22	DUB	*β*-catenin	5-fluorouracil	Resistant	Colorectal cancer	Stemness	[252]
USP10	DUB	YAP	Chemotherapy	Resistant	Breast cancer	Stemness	[256]
USP17	DUB	NRF2	Camptothecin and paclitaxel	Resistant	Colorectal cancer	ROS signaling	[292]
USP15	DUB	KEAP1	Chemotherapy	Sensitive	Multiple cancer types	ROS signaling	[271, 272]
USP29	DUB	HIF-1*α*	Sorafenib	Resistant	Hepatocellular carcinoma	Hypoxia	[287]
Hsc70	Chaperone	HOXB13	Tamoxifen	Sensitive	Breast cancer	Microenvironment	[281]
Hsc70	Chaperone	MORC2	Antiestrogen therapies	Sensitive	Breast cancer	DNA repair	[124]

**Table 2:** Representative small molecules targeting protein degradation under clinical evaluation

Small molecules	Target	Application	Stage of development	Refs.
KSQ-4279	USP1	Patients with advanced solid tumors alone or in combination with an oral PARPi	Phase I	NCT05240898
Perifosine	UCHL3	Refractory tumors	Phase I or II	NCT00873457, NCT01049841, NCT00391560, NCT01097018, NCT00054145
VLX1570	UCHL and USP14	Multiple myeloma	Phase II	NCT02372240
AMG-232	MDM2	Treating patients with acute myeloid leukemia that has come back (recurrent), does not respond to treatment (refractory), or is newly diagnosed in combination with decitabine	Phase I	NCT03041688
ALRN-6924	MDM2/MDMX	Treatment for resistant (refractory) pediatric cancer	Phase I	NCT03654716
DS-3032b	MDM2	Treatment of relapsed and/or refractory multiple myeloma	Phase I	NCT02579824
RO6839921	MDM2	Patients with advanced cancers	Phase I	NCT02098967, NCT01462175
BI 907828	MDM2	Different types of advanced cancer	Phase I	NCT03449381, NCT05376800, NCT03964233
NX-1607	Cbl-b	Advanced malignancies	Phase I	NCT05107674
KPG-818	CRL4	Hematological malignancies	Phase I	NCT04283097

### Targeting proteasomal degradation

#### Small molecules

The role of the ubiquitin–proteasome system in drug resistance has long been considered closely related to cell survival and response to stress [294]. Bortezomib, a proteasome inhibitor, has been approved by the Food and Drug Administration for the treatment of multiple types of tumors since 2003 [295]. Together with the second generation of proteasome inhibitors (e.g., ixazomib, delanzomib, carfilzomib, and oprozomib), proteasome inhibitors potentiate antitumor effects against several tumors [296]. However, proteasome inhibitor resistance may occur in refractory tumors due to the lack of specific targeting ability [297]. Due to the substrate-specific selectivity of DUBs, efforts have been devoted to the development of small molecules targeting DUBs for cancer therapy [35]. Encouragingly, VLX1570, a small molecule targeting UCHL and USP14 is under clinical evaluation for treating multiple myeloma (NCT02372240) [298]. In addition, the small molecule perifosine targeting UCHL3 has been evaluated for the treatment of refractory tumors, including chronic lymphocytic leukemia (NCT00873457) [299], recurrent pediatric solid tumors (NCT01049841) [300], refractory and relapsed leukemia (NCT00391560) [301], refractory advanced colorectal cancer (NCT01097018) [302], and recurrent, refractory, locally advanced, or metastatic breast cancer (NCT00054145) [303]. Moreover, KSQ-4279 inhibits USP1 to effectively cure patients with advanced solid tumors alone or in combination with an oral PARPi, which has entered a phase I clinical trial (NCT05240898). Despite tremendous progress in the development of drugs targeting E1 enzymes [29] and E2 enzymes [30], E3 ligases have better application prospects for precise targeting due to their substrate specificity with approximately 600 members [44]. Notably, many small molecules targeting E3 ligases have been developed to surmount cancer drug resistance [304, 305]. Of note, small molecules targeting MDM2, including AMG-232 (NCT03041688) [306], ALRN-6924 (NCT03654716) [307], DS-3032b (NCT02579824) [308], RO6839921 (NCT02098967, NCT01462175) [309], BI 907,828 (NCT03449381, NCT05376800, NCT03964233) [310], are currently under clinical evaluations for treating refractory or advanced tumors. NX-1607 targeting Cbl-b is in phase I clinical trial for treating advanced malignancies (NCT05107674) [311]. In addition, KPG-818 treating hematological malignancies through targeting CRL4 is also under phase I clinical evaluation (NCT04283097) [312].

#### PROTAC

Despite the rapid development of studies on molecular mechanisms, some critical proteins in cancer cells are still “undruggable” due to the lack of targeted small molecule or E3 ligases [313]. In addition, there is still not a strict one-to-one correspondence between E3 and substrate, suggesting a natural defect in its targeting ability. Hence, proteolysis-targeting chimeras (PROTACs), which consist of two covalently linked protein-binding molecules targeting E3 ligase together with the protein of interest, have been developed to broaden the range of applications and improve the ability to precisely interact with E3 ligases and target proteins [314, 315]. PROTAC technology is based on a clear degradation mechanism to “hijack” the ubiquitin–proteasome system, resulting in rapid and specific degradation of indicated proteins. In 2019, two small molecules (ARV-110 (NCT03888612) and ARV-471 (NCT04072952)) that can be administered orally have entered clinical trials for the treatment of metastatic castration-resistant prostate cancer and advanced breast cancer, respectively. To date, at least 16 PROTAC molecules have entered clinical evaluation for the treatment of various tumors (Table 3).

**Table 3:** Representative PROTACs under clinical evaluation

Names	Targets	E3 ligases	Indications	Status	Refs.
AR-LDD	AR	CRBN	Prostate cancer	Phase I	NCT04428788
ARV-110	AR	CRBN	Prostate cancer	Phase II	NCT05177042, NCT03888612
ARV-471	ER	CRBN	Breast cancer	Phase II	NCT05501769, NCT05463952, NCT04072952
ARV-766	AR	VHL	Prostate cancer	Phase I	NCT05067140
BGB-16673	BTK	Undisclosed	B cell malignancies	Phase I	NCT05294731, NCT05006716
CFT8634	BRD9	CRBN	Synovial sarcoma	Phase I/II	NCT05355753
DT-2216	BCL-XL	VHL	Liquid and solid tumors	Phase I	NCT04886622
FHD-609	BRD9	Undisclosed	Synovial sarcoma	Phase I	NCT04965753
GT20029	AR	Undisclosed	Androgenetic alopecia and acne	Phase I	NCT05428449
HSK29116	BTK	Undisclosed	B cell malignancies	Phase I	NCT04861779
KT-333	STAT3	Undisclosed	Liquid and solid tumors	Phase I	NCT05225584
KT-413	IRAK4	CRBN	B cell lymphomas	Phase I	NCT05233033
KT-474	IRAK4	Undisclosed	Atopic dermatitis and hidradenitis suppurativa	Phase I	NCT04772885
LNK01002	RAS GTPase	Undisclosed	Acute myeloid leukemia	Phase I	NCT04896112
NX-2127	BTK	CRBN	B cell malignancies	Phase I	NCT04830137
NX-5948	BTK	CRBN	B cell malignancies and autoimmune diseases	Phase I	NCT05131022

#### Other approaches targeting proteasomal degradation

Molecular glues are considered a category of small molecules that stabilize the interaction between two proteins [316]. To facilitate targeted protein degradation, molecular glues such as immunomodulatory imide drugs (IMiD, e.g., thalidomide, lenalidomide, pomalidomide) generate a novel interaction between a substrate (e.g., IKZF 1/3) [317, 318] and cereblon, a substrate receptor for CRL4 [319, 320]. Although the underlying molecular mechanisms still need further exploration, thalidomide has already been approved to treat patients with leprosy in 1975, multiple myeloma in 1998; lenalidomide has been approved for the treatment of multiple myeloma in 2006 as well as myelodysplastic syndrome, lymphoma, follicular lymphoma; pomalidomide has been approved in 2013 as a treatment for relapsed and refractory multiple myeloma [321, 322]. In addition, thalidomide (NCT00016224), lenalidomide (NCT01246557, NCT00988208) [323, 324], and pomalidomide (NCT01324947, NCT01311687) [325, 326] are currently under clinical trial for the treatment of resistant and refractory tumors.

As mentioned above, IAPs may be upregulated in refractory tumors due to their biological function of inhibiting apoptosis [327]. Similar to PROTAC, specific non-genetic IAP-based protein erasers (SNIPERs) consist of three structures, including IAP recognition structure, specific ligand for the target protein, and linker [328]. Under physiological conditions, IAP exerts its anti-apoptotic function by ubiquitinating and degrading caspase [329]. SNIPERs utilize the E3 activity of IAP to ubiquitinate and degrade the target protein by linking IAP with the target protein. The specific molecular mechanism remains to be further explored.

In addition, Trim-Away is another approach that targets protein degradation based on the high affinity of TRIM21 to antibodies [330]. TRIM21 binds to the target protein through a specific antibody to form a complex, then TRIM21 is ubiquitinated, and the entire complex is degraded through the ubiquitin–proteasome pathway [331]. Trim-Away has the advantages of a wide range of target proteins, simple and rapid technology, but due to the lack of clinical drug delivery methods, it is currently used for the mechanistic study of cell models or model organisms.

### Targeting lysosomal degradation

#### Small molecules

Autophagy has long been considered a recycling process for coping with intracellular stress during cancer progression [332]. Much evidence indicates that autophagy is an important reason for tumor resistance [333]. Chloroquine, an autophagy‒lysosome pathway inhibitor, is currently under clinical evaluation for treating autophagy-dependent pancreatic cancer (NCT01506973) [334]. However, due to the toxicity of chloroquine and the lack of precise targeting, its clinical application is limited. In addition, autophagy plays a dual role in tumorigenesis and development, and simply inhibiting non-selective autophagy may have side effects on tumor therapy [335].

### Growing approaches targeting lysosomal proteolysis

Similar to the precise targeting of proteasomal proteolysis, several technologies targeting protein degradation through lysosomal proteolysis have emerged spontaneously, including autophagy-targeting chimera (AUTAC), newly developed AUTOTAC, autophagosome-tethering compound (ATTEC), CMA-targeting motif (CTM)-directed protein degradation, and lysosome-targeting chimera (LYTAC). The design of AUTAC stems from the ubiquitin-dependent selective degradation of group A *Streptococcus* (GAS) by autophagy, although the specific E3 ligase has not been identified [336]. AUTOTAC is a category of small molecules connecting the protein of interest and p62, resulting in selective degradation through macroautophagy in a ubiquitin-independent way [337]. Similarly, ATTEC is a molecule that connects the protein of interest and LC3 for macroautophagy-induced degradation [338]. By designing a targeted peptide containing a CTM protein-binding domain interacting with the protein of interest, CTM-directed protein degradation can rapidly degrade endogenous proteins through chaperone-mediated autophagy [339]. LYTAC is designed to traffic secreted or membrane-associated proteins into lysosomes for degradation through the interaction between a specific antibody and a glycopolypeptide ligand for CI-M6PR [340]. At present, small molecules targeting EGFR and integrins have undergone preliminary validation and show clinical potential for the treatment of refractory tumors. [341].

## Conclusions and perspectives

In this review, we have systematically summarized recent studies on protein degradation for regulating cancer drug resistance. Intriguingly, both main degradation pathways, proteasomal proteolysis and lysosomal proteolysis, participate in the regulation of drug resistance, where ubiquitin plays essential roles in the selective degradation of key regulators. Due to their strong substrate specificity, the mechanism by which E3 and DUB regulate the ubiquitination and degradation of key molecules has been deeply explored, and corresponding small molecule inhibitors are being developed to reverse tumor drug resistance. In addition, several ubiquitin-independent selective proteolysis mechanisms, such as chaperone-mediated autophagy, have also attracted significant research interest. However, due to the complexity of the molecular mechanisms and the limited substrates that have been extensively studied, some novel new small-molecule compounds based on the connection between the “undruggable” substrates and the key regulators in proteolysis have been developed and have achieved gratifying results in clinical applications.

The important impact of protein degradation regulation on drug resistance is often overlooked in the typical research process. In-depth and systematic study of the protein degradation mechanism can help improve the understanding of drug resistance and provide new targets and therapeutic strategies for drug development. In addition, traditional small molecules inhibit the enzymatic activity of the target by binding to the target protein for a long period, which remains challenging for both the structural design of small molecules and the target mutation-mediated drug resistance. By designing small molecules (e.g., PROTAC) that only require a short-term connection between the target protein and a specific key regulator of protein degradation, the target protein can be selectively degraded, which facilitates drug development and improves the killing effect on mutant tumors.

Although some research progress has been made on ubiquitination and deubiquitylation, the current research on ubiquitination is mainly focused on the proteasome pathway, while studies on the upstream ubiquitin regulation of the lysosomal degradation pathway are still limited. Among the current technologies targeting protein degradation, the molecular mechanism of PROTAC has been researched in-depth, while the others still need further study on the underlying molecular mechanisms, which may facilitate the clinical use of other technologies targeting protein degradation. In summary, continuing to further explore the regulatory mechanisms of ubiquitin-dependent and ubiquitin-independent selective degradation and clarify its regulatory relationship with tumor drug resistance will provide meaningful guidance for the clinical treatment of tumor drug resistance.

In addition to regulating genes and RNAs, targeted protein degradation has become a hot research topic due to its advantages of direct and fast action. With the development of multiomics technology, understanding the ubiquitin code that regulates key molecules of tumor resistance from a mechanistic perspective will promote the in-depth understanding and application of protein degradation mechanisms. Additionally, several ubiquitin-independent protein degradation pathways should also take into consideration for further research. Studying the interaction between proteasomal proteolysis and lysosomal proteolysis in mediating the degradation of tumor resistance-related molecules will provide a reasonable route for the development of technologies that selectively target protein degradation. Structural optimization of synthetic molecules designed for different degradation pathways will promote the specificity of degradation, reduce biotoxicity and further clinical applications. In addition, as an important part of the protein expression regulation mechanism, protein degradation should also be coordinated with research on gene expression, RNA modification, and other protein modifications, acting as a supplement to overcome the acquired drug resistance mechanism and offering new strategies to surmount cancer drug resistance.

## Data Availability

Not applicable.

## Abbreviations

53BP1: P53-binding protein 1
6-TG: 6-Thioguanine
*β*-TrCP: *β*-Transducin repeat-containing protein
ABC: ATP-binding cassette
AhR: Aryl hydrocarbon receptor
AMFR: Autocrine motility factor receptor
AML: Acute myeloid leukemia
AMPK-*α*1: AMP-activated protein kinase *α*1
ANAPC2: Anaphase-promoting complex subunit 2
APAF-1: Apoptotic protease activating factor 1
ASPM: Abnormal spindle-like microcephaly-associated protein
ATTEC: Autophagosome-tethering compound
AUTAC: Autophagy-targeting chimera
BCA2: Breast cancer-associated gene 2
BCL-2: B cell lymphoma 2
BCRP: Breast cancer resistance protein
BET: Bromodomain and extra-terminal
BIRC3: Baculoviral IAP repeat-containing 3
BRCA1: Breast cancer type 1 susceptibility protein
Cbl-b: Casitas b-lineage lymphoma-b
CDC25A: Cell division cycle 25 A
CDK: Cyclin-dependent kinase
CGRRF1: Cell growth regulator with RING finger domain protein 1
CHIP: C-terminus of HSC70-interacting protein
CHK: Checkpoint kinase
CRL4: Cullin-RING ubiquitin ligases 4
CYP450: Cytochrome P450 enzymes
CMA: Chaperone-mediated autophagy
CSC: Cancer stem cell
CTM: CMA-targeting motif
CYLD: Cylindromatosis-associated DUB
CYPA: Cyclophilin A
DAB2IP: DOC-2/DAB2 interactive protein
DDR: DNA damage response
DNA-PKcs: DNA-dependent protein kinase catalytic subunit
DSB: DNA double-strand break
DUB: Deubiquitylating enzyme
EGFR: Epidermal growth factor receptor
EMT: Epithelial-to-mesenchymal transition
ERAD: Endoplasmic reticulum-associated degradation
ERK: Extracellular signal-regulated kinase
FLIP: FLICE-inhibitory protein
GAS: Group A *Streptococcus*
GBM: Glioblastoma
GDP: Guanosine diphosphate
GSK3*β*: Glycogen synthase kinase 3*β*
GST: Glutathione S-transferase
HBXIP: Hepatitis B virus X-interacting protein
HCC: Hepatocellular carcinoma
HECT: Homologous to the E6AP C-terminus
HMGCoAR: 3-Hydroxy-3-methylglutaryl-coenzyme A reductase
HOXB13: Homeobox B13
HIF-1*α*: Hypoxia-inducible factor 1 alpha
HR: Homologous recombination
IAPs: Inhibitors of apoptosis proteins
IKK: Inhibitor of NF-*κ*B kinase
JAK: Janus kinase
JNK: C-Jun *N*-terminal kinase
KEAP1: Kelch-like ECH-associated protein 1
KRAS: Kirsten rat sarcoma
LAMP2A: Lysosome-associated membrane protein type 2A
LSCC: Lung squamous cell carcinoma
LUAD: Lung adenocarcinoma
LYTAC: Lysosome-targeting chimera
LZTR1: Leucine zipper-like transcriptional regulator 1
MAPK: Mitogen‑activated protein kinase
MARCH: Membrane-associated RING-CH
MDC1: Mediator of DNA damage checkpoint protein 1
MDM2: Mouse double minute 2 homolog
MAST: Microtubule-associated serine/threonine kinase 1
MDR: Multi-drug resistance
MKP1: Mitogen-activated protein kinase phosphatase-1
MRP: MDR-associated protein
MORC2: Microrchidia family CW-type zinc finger 2
MM: Multiple myeloma
MMR: Mismatch repair
MSH2: MutS protein homolog 2
MT: Methyltransferase
MTAP: Methylthioadenosine phosphorylase
NAT: *N*-Acetyltransferase
NEDD4L: Neural precursor cell expressed developmentally downregulated gene 4-like
NHEJ: Nonhomologous end-joining
NPC: Nasopharyngeal carcinoma
NRF2: Nuclear factor erythroid 2-related factor 2
NSCLC: Non-small-cell lung carcinoma
PARP: Poly (ADP-ribose) polymerases
PBX1: Pre-B-cell leukemia homeobox-1
PCa: Prostate cancer
PI3K: Phosphoinositide 3-kinase
PLK: Polo-like kinase
PRKACA: Protein kinase cAMP-activated catalytic subunit alpha
PP2A: Protein phosphatase 2A
PRMT5: Protein arginine methyltransferase 5
PROTAC: Proteolysis targeting chimera
PUMA: P53 upregulated modulator of apoptosis
pVHL: Von Hippel–Lindau protein
RBX1: DOC-2/DAB2 interactive protein 1
RBR: RING-between-RING
RING: Really interesting new gene
ROS: Reactive oxygen species
SCF: SKP1/CUL1/F-box
SGCE: Sarcoglycan epsilon
SH3RF1: SH3 domain-containing RING finger 1
SIRT1: Sirtuin 1
SKP1: S-phase kinase-associated protein 1
SMURF2: Smad ubiquitination regulatory factor 2
SNIPERs: Specific non-genetic IAP-based protein erasers
SOCS5: Suppressor of cytokine signaling 5
STAT: Signal transducer and activator of transcription
SULT: Sulfotransferase
STUB1: STIP1 homology and U-Box-containing protein 1
TPD: Targeted protein degradation
TRIM15: Tripartite motif-containing protein 15
TKI: Tyrosine kinase inhibitor
TNBC: Triple-negative breast cancer
UBE2R1: Ubiquitin-conjugating enzyme E2 R1
UCHL3: Ubiquitin C-terminal hydrolase L3
UGT: UDP-glucuronosyltransferase
USP: Ubiquitin-specific peptidase
UV: Ultraviolet
VCP: Valosin-containing protein
XRCC4: X-ray repair cross-complementing protein 4
YAP: Yes-associated protein
